# Olive fruit volatiles route intraspecific interactions and chemotaxis in *Bactrocera oleae* (Rossi) (Diptera: Tephritidae) females

**DOI:** 10.1038/s41598-020-58379-8

**Published:** 2020-02-03

**Authors:** Giulia Giunti, Orlando Campolo, Francesca Laudani, Giuseppe Massimo Algeri, Vincenzo Palmeri

**Affiliations:** Department of Agriculture, University “Mediterranea” of Reggio Calabria, Loc. Feo di Vito, 89122 Reggio Calabria, Italy

**Keywords:** Behavioural ecology, Animal behaviour

## Abstract

Plant nutritional quality and chemical characteristics may affect the fitness of phytophagous insects. Here, the olfactory preferences of *Bactrocera oleae* (Rossi) females toward olives with different maturation and infestation status were evaluated in three cultivars: Ottobratica, Roggianella and Sinopolese. Volatile profiles from olives were identified by SPME/GC-MS. Choice tests were performed to determine the responses of *B. oleae* adult females toward fruits and pure chemicals linked to infestation degree. Cultivar was the main source of variability explaining the differences recorded in volatile emissions. Moreover, three VOCs [*β*-myrcene, limonene and (*E*)-*β*-ocimene] were associated to infestation status across all olive varieties. In choice-tests, *B. oleae* females always preferred the olfactory cues from low-infested over high-infested fruits. Therefore, choice-tests using synthetic VOCs, emitted in greater amount by high-infested fruit, were arranged in order to identify putative *B. oleae* kairomones. While females were indifferent to *β*-myrcene, the highest dosages of limonene and (*E*)-*β*-ocimene were unfavoured by the tested flies, which preferentially moved toward the empty arm of the Y-tube. Furthermore, females preferred the lowest concentration of *β*-ocimene compared to the highest one. These results supported our hypothesis that fruit VOCs may serve as kairomones for female flies.

## Introduction

Environmental conditions, food availability and ovipositional sites are crucial for phytophagous-insect survival^1,2^. The nutritional quality and the attractiveness of plants may influence the fitness of herbivores and be a key factor for oligophagous species, which can rely on a restricted number of host-plants^3,4^. Therefore, the recognition of suitable ovipositional sites is critical for several fruit flies^5,6^.

*Bactrocera oleae* (Rossi) (Diptera: Tephritidae), commonly known as the olive fruit fly, is a strictly monophagous pest, which can feed exclusively on *Olea* species. The larval stages feed and develop solely inside olive drupes, excavating galleries inside the pulp. The fully developed larvae open an exit hole on the olive surface and usually pupate outside the fruit. During the oviposition process, *B. oleae* females carefully choose suitable fruit for oviposition, either performing aggressive behaviours toward conspecifics to gain and maintain the oviposition site^7^. In the Mediterranean area, the olive fruit fly population commonly increases during summer season until the harvest period. Depending on climate conditions, *B. oleae* produce from 2 to 7 generations during the favourable season, although this fly can reproduce also during winter in mild climates^8^. The olive fruit fly is considered the key pest of olive crop (*Olea europea* L.), causing great qualitative and quantitative yield loses, as well as premature fruit drop. The presence of ovipositional puncture and/or active infestation can decrease or even nullify the value of table olives and seriously reduce oil yield and quality^9,10^. Furthermore, the olive fruit fly can act as vehicle of phytopathogenic agents, which can heavily increase crop loses^11^.

In the Mediterranean basin, the olive tree is an historical and traditional crop. In the last decade, the global olive and olive oil production has increased consistently, as this cultivation has been adopted in novel areas, such as South America and Eastern Asia. *Bactrocera oleae* has followed the extension of olive growing becoming an economic problem worldwide^12^. Although chemical control remains the main strategy against this pest, the occurrence of resistant insect strains^13,14^, the inconstant efficacy of chemical treatments^12^, as well as the increasing awareness of the adverse environmental side-effects of pesticides^15^, have emphasised the need to develop innovative approaches to control *B. oleae*. In this scenario, the promising results using semiochemicals in laboratory and controlled conditions suggest the potential of their introduction in IPM programs^16^.

Susceptibility to insect-pest infestation results primary from the ovipositional preferences of mated females and secondary from the optimal larval foraging substrate. For *B. oleae*, host-plant quality is known to vary both among the *Olea* species as well as among *O. europaea* cultivars^17^, although also abiotic factors (i.e. light intensity) and microorganisms could alter the egg laying and production by female flies^18,19^. Aside from female oviposition preferences, fruit characteristics may influence the larval development^4^. Among fruit features, the ripening stage influences *B. oleae* oviposition choices, since females generally prefer to oviposit on unripe green olives^4,6^. To successfully locate and select oviposition sites, *B. oleae* adult females mainly rely on olfactory cues, namely volatile organic compounds (VOCs, hereafter) produced by the tree^20^. Either biotic and abiotic factors can modify the VOC emissions from olives^21,22^ and can change their attractiveness toward the olive fruit fly and its natural enemies^23,24^. As reported for other phytophagous species^25,26^, the presence of a pre-existing infestation can alter the volatile emission and can decrease fruit appeal for conspecific oviposition, reducing progeny competition during the larval development.

To date, no conclusive information is available on the innate chemotaxis of *B. oleae* females according to the incidence of conspecific infestation. It has been documented that *B. oleae* females, just after egg laying, regurgitated small droplets of olive juice around the oviposition site deterring other female flies to oviposit on the same fruit^27^. Moreover, *B. oleae* females showed positive chemotaxis toward intact olives, while repellence activity was reported for volatiles produced by mechanically damaged fruits^28^. To date, no information is reported on the ability of *B. oleae* females to quantify the incidence of conspecific competitors by exploiting the olfactory cues produced by the fruit. A single olive drupe can sustain the development of multiple larvae (i.e. two larvae in average, depending on fruit dimensions)^3^, allowing *B. oleae* females to oviposit also on previously-infested fruit, although the presence of prior infestation can cause fruit depletion and unsuitability for oviposition.

In this research, we investigated, under laboratory conditions, the impact of olfactory cues on the selection of potential ovipositional sites by female flies, with particular concern to infestation severity. We hypothesize that the VOCs produced by the olives may play a role on *B. oleae* searching behaviour, allowing females to select suitable oviposition sites. Since ripening has been documented to influence the oviposition choices, the maturation degree of fruit has been also considered. The olives from three different cultivars were tested. The selected olive varieties (cv. Ottobratica, cv. Roggianella and cv. Sinopolese) are traditionally cultivated in southern Italy, and they are among the most economically important in Calabria region. Furthermore, the volatile emissions produced by these cultivars were never examined previously. Firstly, we evaluated females’ preferences in two-choice bioassay comparing, within the cultivars, the attractiveness of olives with different maturation degree or infestation status. Subsequently, volatiles emitted by olives were SPME-sampled and analysed by gas chromatography-mass spectrometry (GC-MS) to estimate emissions attributable to cultivar peculiarities, maturation degree and infestation status. A multivariate statistical approach was applied to determine the occurrence of VOCs commonly or exclusively produced by the tested cultivars as a consequence of ripening and pest incidence, as well as to detect putative herbivorous-induced plant volatiles (HIPVs). Lastly, synthetic chemical compounds differentially emitted according to infestation degree were used in two-choice bioassay to assess *B. oleae* responses and to detect putative kairomones. The pure compounds were tested at different concentrations to assess possible dose-dependent behavioural responses by mature female flies.

## Results

### Preferences of *B. oleae* females toward olive fruits

**Figure 1:**
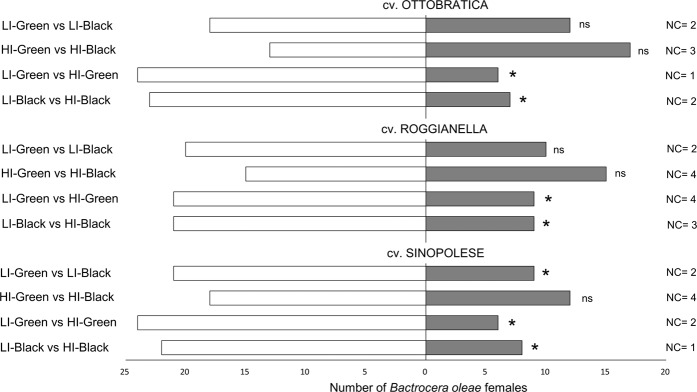
Behavioural responses of *Bactrocera oleae* females towards olive fruits: effect of maturation degree and infestation status. Two-choice bioassays were conducted in Y-tube with olive fruit providing olfactory stimuli. Thirty flies were tested for every comparison. For every bioassay, asterisks indicate significant differences in the number of flies choosing different cues (*χ*^2^ test with Yates correction, *P* < 0.05). LI = low-infested fruit; HI = High-infested fruit; NC = no-choice flies; ns = not significant.

*Bactrocera oleae* females showed significant preferences for low-infested (LI) over high-infested (HI) fruits (Fig. 1). Female flies preferred LI fruit regardless the maturation degrees of the tested olives, i.e. black (Ottobratica: *χ*^2^ = 8.57, df = 1, *P* = 0.003; Roggianella: *χ*^2^ = 4.83, df = 1, *P* = 0.028; Sinopolese: *χ*^2^ = 6.57, df = 1, *P* = 0.010) and green (Ottobratica: *χ*^2^ = 10.83, df = 1, *P* = 0.001; Roggianella: *χ*^2^ = 4.83, df = 1, *P* = 0.028; Sinopolese: *χ*^2^ = 10.83, df = 1, *P* = 0.001). Flies did not show any preferences between green and black olives either at low or high infestation status for the cultivars Ottobratica (HI Olives: *χ*^2^ = 0.57, df = 1, *P* = 0.567; LI Olives: *χ*^2^ = 1.23, df = 1, *P* = 0.267) and Roggianella (HI Olives: *χ*^2^ = 0.03, df = 1, *P* = 0.855; LI Olives: *χ*^2^ = 3.37, df = 1, *P* = 0.067). When testing Sinopolese olives, no preference was recorded between black and green HI olives (*χ*^2^ = 1.23, df = 1, *P* = 0.267), but LI-Green fruits were significantly more attractive for *B. oleae* females than LI-Black ones (*χ*^2^ = 4.83, df = 1, *P* = 0.028). Concerning latent periods, no significant difference was recorded for the treatments and the cultivars tested (Supplementary Table S1).

### Effect of *B. oleae* infestation and fruit maturation on VOC emission

GC-MS analyses identified over 70 different volatile compounds. VOC bouquet qualitatively varied according to the tested cultivar. Fifty-eight compounds were collected and identified from Ottobratica fruit, 54 from Sinopolese olives and only 51 from Roggianella ones. Seven volatiles [1-dodecene, (*Z*)-6-tridecene, 10-undecenal, tetradecanal, *α*-longipinene, dihydro-*β*-agarofuran, longipinanol] were exclusively produced by Ottobratica fruit, while cv. Sinopolese presented only 3 exclusive VOCs [n-nonadecane, n-eicosane, liguloxide]. Roggianella did not present exclusive volatiles. Moreover, (*E*)-*β*-farnesene is absent only in cv. Ottobratica and (*E*)-2-Nonen-1-ol in cv. Sinopolese olives.

The tested cultivars showed differential emissions attributable to infestation status and maturation degree. The two factors General Linear Model (GLM) indicated that in Ottobratica and Sinopolese olives a high number of VOCs (33 for Ottobratica and 22 for Sinopolese) significantly varied as a result of maturation and/or infestation status (Supplementary Tables S2 and S3). In contrast, cv. Roggianella slightly modified VOC emission (11 compounds) according to the considered variables (Supplementary Table 4). All the tested cultivars emitted 3 monoterpenes, which were prevalently produced by high-infested olives: β-myrcene, limonene and (*E*)-β-ocimene. Furthermore, Otttobratica and Sinopolese olives released 8 additional shared VOCs correlated to infestation severity (*α*-terpinene, *o*-cymene, *γ*-terpinene, *α*-terpinolene, nonanal, (*Z*)-*α*-bergamotene, valencene and (*E,E*)-*α*-farnesene) and 3 shared compounds related to maturation status (hexyl acetate, 2-methyl-6-methylene-1,7-octadien-3-one and *α*-muurulene), which were absent or not significantly different among Roggianella samples. Nevertheless, the sesquiterpene (*E*)-*β*-farnesene was found to be more incipient in green fruit and to be positively related to low-infestation in both Roggianella and Sinopolese fruits. Among chemical classes, monoterpenes hydrocarbons increased according to infestation in cv. Ottobratica and cv. Sinopolese, while hydrocarbons were emitted in greater amount by black olives in cv. Sinopolese and cv. Roggianella.

**Figure 2:**
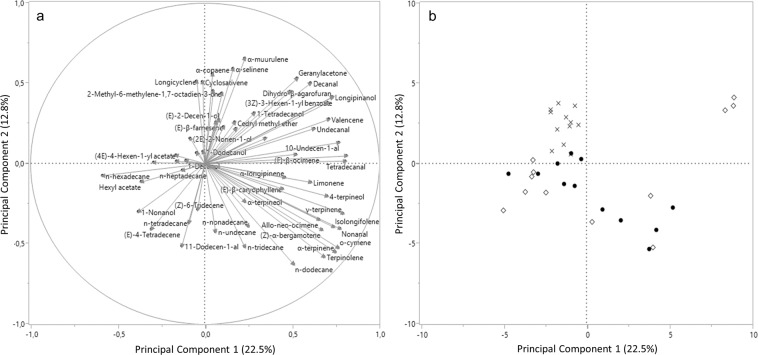
Principal Component Analysis (PCA) of volatile profiles olive fruits of three different olive cultivars. (**a)** PCA loading plot, showing volatile connection to the first and second principal component; (**b)** PCA score plot, highlighting cluster of volatiles attributable to cultivar. ◊ Ottobratica; х Roggianella; ● Sinopolese.

PCA followed by discriminant analysis highlighted differences between volatile emissions from olive fruit attributable to cultivar, maturation and infestation. Since no correlations between the majorities of the compounds occurred (i.e. the variables could be considered independent), the Kaiser coefficient was around 1.00. Eight principal components, explaining 71.75% of variation, were selected and analysed (Supplementary Table S5). PCA results are presented in Fig. 2a. A two-dimensional score plot was also performed to allow easier identification of source of variation considering the first and the second principal component (Fig. 2b). Similar two-dimensional plots were also developed for each cultivar, to enlighten possible variations attributable to infestation status or maturation degree. Supplementary Fig. S1 shows how, within every cultivar, the infestation status plays a key role as source of variation, although slightly for cv. Roggianella. For every single VOC, the eigenvectors are presented in Supplementary Table S6 and the rotated factors in Supplementary Table S7. Based on GLM results, the evaluated factors (n = 8) were labelled as: Factor1 “cv. Ottobratica & cv. Sinopolese”, Factor2 “cv. Ottobratica”, Factor3 “Infestation status”, Factor4 “cv. Roggianella”, Factor5 “Infestation status cv. Roggianella”, Factor6 “Infestation status*Maturation”, Factor7 “Green” and Factor8 “Black” (Supplementary Table S8). Concerning discriminant analysis, the Wilks Lambda test showed a *P* value < 0.0001 and no misclassified variables were recorded for all the tested factors. Step-wise method emphasized 10 variables associated to infestation status and 15 variables highly correlated to maturation degree. Infestation status can be described by 4 chemicals emitted typically by fruit with low *B. oleae*-infestation, and 6 highly correlated to high-infested fruit (Table 1). Among the volatiles highly correlated with maturation, the majority are associated with green olives while 5 VOCs with black olives (Table 2).

**Table 1:** Volatiles identified after discriminant analysis using “infestation status” as category. Positive correlations with Canonical1 indicate VOCs associated to *Bactrocera oleae* high-infestation, while negative correlations represent volatiles prevalently emitted by low-infested olives.

Compound	Correlation with Canonical 1
*β*-myrcene	0.5902931
*o*-cymene	0.438327
Limonene	0.2850285
(*E*)-*β*-ocimene	0.320211
(2*E*)-2-Nonen-1-ol	−0.488669
11-Dodecenal	−0.366617
(*E*)-β-farnesene	−0.6342
Valencene	0.5836008
*α*-selinene	0.3352083
*α*-muurulene	−0.661591

**Table 2:** Volatiles identified after discriminant analysis using “maturation degree” as category. Positive correlations with Canonical1 indicate VOCs associated to green fruits, while negative correlations represent volatiles prevalently emitted by black olives.

Compound	Correlation with Canonical 1
Hexyl acetate	−0.030095
*α*-terpinene	0.1834896
2-Methyl-6-methylene-1,7-octadien-3-one	0.6503437
*trans-*alloocimene	0.001052
*α*-terpineol	−0.016267
*n*-dodecane	0.1578933
(2*E*)-2-Decen-1-ol	−0.040345
(6*Z*)-6-Tridecene	0.0501884
(*E*)-*β*-farnesene	−0.067958
*α*-muurulene	0.1598099
*β*-selinene	0.0867047
(*E,E*)-*α*-farnesene	−0.340786
Longipinanol	−0.346691
Epi-cedrol	0.0591797
*n*-nonadecane	0.2153667

### Behavioural responses of *B. oleae* females toward selected synthetic VOCs

**Figure 3:**
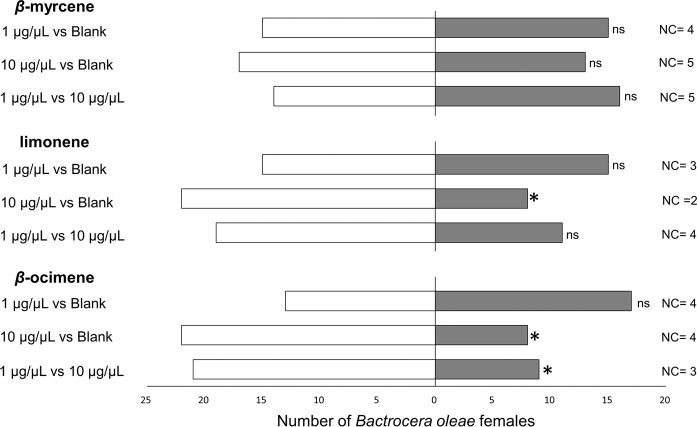
Y-tube choice-tests evaluating the olfactory preferences of *Bactrocera oleae* females for olive VOCs linked to fruit infestation status. Every synthetic chemical was diluted in n-hexane at 1 or 10 μg/μL. For bioassays, 5 μL of n-hexane solution were tested. Thirty flies were tested for every comparison. For every bioassay, asterisks indicate significant differences in the number of flies choosing different cue (χ^2^ test with Yates correction, *P* < 0.05). Blank = 5 μL of n-hexane; NC = no-choice flies; ns = not significant.

According to the results presented above from two-choice bioassays with drupes, *B. oleae* females preferred the olfactory cues from low-infested olives for all the tested cultivar. Since three monoterpenes, *β*-myrcene, limonene and (*E*)-*β*-ocimene, were prevalently produced by high-infested olives in all the tested cultivars, Y-tube bioassays at different dosages of pure chemicals were performed. Overall, some anemotaxis was documented for *B. oleae*, since a low number of flies did not move in the olfactometer and the active flies also moved toward the non-odorized arm of the Y-tube (Fig. 3). Pure *β*-myrcene had no effect on *B. oleae* orientation at either test concentration (1 μg/μL: *χ*^2^ = 0.03, df = 1, *P* = 0.855; 10 μg/μL: *χ*^2^ = 0.57, df = 1, *P* = 0.452), nor when the two different dosages were compared (*χ*^2^ = 0.17, df = 1, *P* = 0.683). At the lowest tested concentration (i.e. 1 μg/μL), female olive fruit flies were also indifferent to limonene: (*χ*^2^ = 0.03, df = 1, *P* = 0.855) and *β*-ocimene (*χ*2 = 0.57, df = 1, *P* = 0.451). Conversely, at the highest concentration (10 μg/μL), limonene and β-ocimene were unfavoured by *B. oleae* adult females compared to blank (limonene: *χ*^2^ = 6.57, df = 1, *P* = 0.001; *β*-ocimene: *χ*^2^ = 6.57, df = 1, *P* = 0.001). When comparing in Y-tube different concentrations of pure *β*-ocimene, *B. oleae* preferred the lowest concentrated stimulus (*χ*^2^ = 4.83, df = 1, *P* = 0.028). In contrast, female flies showed no preferences between the two different concentrations of limonene when compared each other in two-choice assays (*χ*^2^ = 2.17, df = 1, *P* = 0.141). No significant difference was found between the latent periods in all trials (Supplementary Table S1).

## Discussion

Fruit characteristics are crucial for the oviposition site selection of *B. oleae*^6,29^. Moreover, the quality of oviposition sites can be deeply altered by exogenous factors, as the presence of prior infestations^5^. Previous studies have demonstrated that the presence of *B. oleae* larvae inside the fruit modifies the volatile emissions of olives compared to uninfested ones, triggering the production of *β*-ocimene^22,23^. Therefore, VOCs associated to *B. oleae* infestation may be exploited by the female flies as kairomones for oviposition-selection. This kind of intraspecific interaction can be defined as plant mediated interaction (i.e. when a pest or a pathogen induces changes in the shared hostplant affecting the activity and the behaviour of the subsequent attackers, whether they are conspecific or heterospecific). This kind of indirect competition is more frequent in interspecific interactions (i.e. involving contending species), but it can be also exploited by conspecifics^30,31^. Intraspecific competition is probably the most triggering kind of competition for monophagous species, since the conspecific individuals share the same resource requirements^25,26^. Here, the results obtained by both behavioural assays and GC-MS analyses support our hypothesis that olfactory cues from olives play a role on the preferences of *B. oleae* females. Indeed, in Y-tube two-choice trials, *B. oleae* females discriminated fruits with high and low infestation of conspecifics and preferred the olives with low infestation. When females lay eggs inside fruit previously infested by conspecifics, their progeny can suffer from developmental failure, thus it is critical for *B. oleae* to detect the quality of the oviposition sites to maximise their fitness outcomes.

The comparison among VOC profiles from the tested cultivars showed that cultivar is the main source of variability. Results from statistical analysis proved that the largest number of Factors (Factor 1, Factor 2, Factor 4 and Factor 5) explains genetic differences and cultivar specificity. As expected, maturation degree influenced VOC emissions, with two Factors (Factor 7 and Factor 8) linked to ripening stage. Furthermore, Factor 3 explained the variability attributable to infestation status among all the tested cultivars. The emission of some volatiles was correlated to both infestation status and maturation, with a general decrease of VOC emissions in high-infested and ripe fruits. Volatile bouquet of cv. Ottobratica was the richest, while cv. Roggianella presented a restricted VOC profile. While cv. Roggianella showed small VOC variations, volatile emissions from Ottobratica and Sinopolese olives were more influenced by infestation status and/or maturation degree. However, fewer VOCs than expected were found to change according to ripening in every cultivar, and no shared compound linked to maturation was identified in all the tested cultivars. This finding is consistent with the behavioural responses of *B. oleae* in two-choice trials with olive fruits. Female flies showed no preferences for olives at different ripening stages for cv. Roggianella and cv. Ottobratica, while they preferred green fruit in cv. Sinopolese just in presence of low pest infestation. This behavioural response may be explained by the peculiar profile of Sinopolese olives compared to the other varieties, that presented specific VOCs associated to unripe fruit. Considering all the tested cultivars, the molecules 2-methyl-6-methylene-1,7-octadien-3-one and *α*-muurulene were generally produced by unripe olives, while hexyl acetate by ripe fruits. Hexyl acetate belongs to the class of C6 esters and it is produced by the lipoxygenase pathway, a metabolomic pathway typically activated by the tree during fruit ripening^32^. The sesquiterpene *α*-muurulene has been already described as an olive constituent linked to unripe fruit^21^, while the ketone 2-methyl-6-methylene-1,7-octadien-3-one has been identified as green leaf volatile (GLV) of some olive species^27^.

The infestation status of the fruit affected the VOC emissions from the drupes and routed the preferences of *B. oleae* females in laboratory trails. Female flies preferred the olfactory stimuli emitted by low-infested olives compared to high-infested fruits, disregarding the tested cultivar or the ripening stage. The identification of volatiles from olives demonstrated that 3 VOCs significantly increased in high-infested fruit from all the tested cultivars and may be responsible of the innate chemotaxis of *B. oleae* recorded in two-choice trails toward fruit odours. In detail, the monoterpenes *β*-myrcene, limonene and (*E*)-*β*-ocimene increased according to infestation in all the evaluated cultivars. Many tephritid flies rely on these monoterpenes for reproductive purposes, such as mating and oviposition behaviour^33–36^. Nevertheless, these volatiles have been already identified as constituents of the volatile profiles of fruits from other olive cultivars^22,23^. Previous researches reported that (*E*)-*β*-ocimene is positively correlated to infestation, and uninfested olives generally produce no or small amounts of this VOC^22,23^. Furthermore, (*E*)-*β*-ocimene serves as HIPV for several braconid parasitoids^37–39^, including the olive fruit fly’s parasitoid *Psyttalia. concolor* (Szépligeti) (Hymenoptera: Braconidae)^23^. Concerning *β*-myrcene and limonene, to the best of our knowledge, this is the first time that these VOCs have been positively related to infestation of olive drupes, although Giunti *et al*.^24^ proved that limonene can act as attractant toward *P. concolor* males. Here, behavioural trials using pure synthetic chemicals were used to detect putative kairomones explaining the innate preferences of *B. oleae* females for olfactory stimuli emitted by olives with low infestation. In two-choice tests, *B. oleae* generally showed anemotactic responses, considering the low number of no-choice recorded and the walking activity toward the non-odorized branch of the olfactometer. Concerning the pure chemicals, *β*-myrcene was indifferent to olive fruit fly females, while limonene and *β*-ocimene were unfavoured by *B. oleae* females at the highest dosages. When comparing different dosages of pure *β*-ocimene, olive fruit fly females preferred the Y-tube arm containing the lowest concentration of chemicals, assisting the hypothesis that this VOC may serve as kairomone for female flies. Nevertheless, here the behavioural responses of female flies toward olive fruits and pure chemicals were recorded under laboratory conditions. Therefore, it is uncertain if these VOCs can be exploited by *B. oleae* also at longer distances in a complex agroecosystem, in presence of a number of other olfactory stimuli. In this scenario, synthetic kairomones may be useful to improve biological control programs in olive orchards^40,41^, although their possible application in field condition should be carefully evaluated.

## Materials and Methods

### Plant material

Olives from three different cultivars (Ottobratica, Roggianella and Sinopolese) were collected from organic olive grove placed in southern Italy, in Calabria region, near the town of Delianuova (38°1458.0“N; 15°5509.8“E), on the slopes of Aspromonte mountain massif (elevation ca. 600 m). Olives used for behavioural assays and GC-MS analyses were collected in November 2017 from non-irrigated 20 years old olive trees. Fruits were yielded manually (ca. 5 Kg for every cultivar), stored into glass jars and transferred to laboratory conditions within 3 hours.

The olives were firstly separated according to the maturation index (MI), whereby the skin and flesh colours were scored to a 0 to 7 scale^42^. Olives with MI from 0 to 1 were scored as “Green”, while the fruits with MI from 3 to 4 were labelled as “Black”. Half-ripe olives (MI = 2) and over-matured fruits with pigmented pulp (MI = 5–7) were discharged from further analyses, as fruits presenting elevated oleuropein levels and high lipidic amount are less attractive for *B. oleae* oviposition, as well as less suitable for larval development^43^. Among both Green and Black olive bulks, two different infestation degrees were selected: low-infested [i.e. olives with 1–2 *B. oleae* oviposition punctures on the epicarp, but no exit holes; LI, hereafter] and high-infested fruits [i.e. olives with 3–4 *B. oleae* oviposition punctures and 1 exit hole; HI, hereafter]. Thus, both low- and high-infested fruits presented an ongoing infestation, but high-infested olives also bore a previous infestation, ended with the pupation of the fully developed larvae outside the fruit. These infestation levels were chosen also considering that the small drupes from oil variety may usually sustain the development of 2 larvae, before rotting and drying out. The remaining olive fruits, which presented different infestation status, were limited in number and thus not considered for further analyses. Similarly, crushed and naturally damaged olives were discarded from GC-MS analyses and behavioural assays.

Due to a particularly high population density during the 2017, uninfested olives were too few to be considered for further analyses. Previous researches demonstrated that olive fruits collected on different months and/or different seasons can produce different volatilome^20,21^. Therefore, we decided not to sample uninfested olives on the following season. Nevertheless, it has been demonstrated that uninfested olives differ from infested drupes in term of *(E)-β*-ocimene emissions^20,22,23^. Although every olive cultivar presents a different volatilome, exclusively the emission of this monoterpene increases in presence of *B. oleae* infestation crosswise to the olive varieties.

Here, the drupes from every cultivar and maturation index were split in four treatments: (i) high-infested black olives (HI-Black); (ii) high-infested green olives (HI-Green); (iii) low-infested black olives (LI-Black) and (iv) low-infested green olives (LI-Green). Fruits from different treatments were stored separately into sealed clean glass jars (diameter 10 cm, length 20 cm), to avoid volatile contaminations. Before being tested for GC-MS analyses or behavioural assays, olives were stored at laboratory conditions (20 ± 1 °C, 45–55% R.H.) for 12–36 hours. All olives used for GC-MS analyses and for bioassays were subsequently dissected to check the effective presence of *B. oleae* larvae inside the fruit.

### Insect colony

*Bactrocera oleae* was reared as described by Canale *et al*.^44^. Pupae of *B. oleae* were obtained from field-collected olives arriving at a pressing plant in Delianuova (RC, Italy) in 2017 from October to November. Pupae (ca 500 per cage) were held in BugDorm-6S610® (MegaView Science Education Services Co., Ltd., Taichung, Taiwan) and maintained under controlled conditions at 22 ± 1 °C, 45–55% RH until adult emergence. Adult flies were maintained in the cages to allow mating. *Bactrocera oleae* adults were fed on a dry diet of hydrolysed yeast and sucrose (1:10 wt:wt), while water was provided ad libitum on a cotton wick.

### Olfactometry

Behavioural assays were conducted at 22 ± 1 °C (45–55% R.H.). The bioassays were performed in a Y-tube olfactometer connected with an air delivery system, equipped with an activated charcoal filter, which blown the purified air at 0.3 L min^−1^ constant flow. The Y-tube system consisted in a horizontal glass unit with a central tube (100 length × 15 mm diam) and two lateral arms (90 length × 15 mm diam) ending with a spherical trap chamber (50 mm diam). The lateral arms of the Y-tube were connected by Teflon tubes to two Drechsel bottles (250 mL), containing the odorous sources. Similarly, the Drechsel bottles were connected to the air delivery system (Sigma Scientific LLC, Micanopy, FL – USA) through Teflon connections. To exclude the presence of visual cues affecting insect orientation from the surroundings, a preliminary trial was performed using no odorant sources (blank vs blank), and no positional effect was recorded. In addition, all the trials were carried out in a white plastic box (1.5 × 1 × 0.7 m) illuminated from above with cold fluorescent tubes (20 W, 250 lux). To avoid for daily variability and positional effect, replicates of every treatment were carried out over several days (4–5 days) and the olfactometer arms were inverted after every test. The olfactometer was cleaned with warm water (35–40 °C) and mild soap and then rinsed with distilled water every 6 replicates (i.e. 6 tested insects).

*Bactrocera oleae* mated females were tested after 14–21 days from eclosion and were tested in bioassays only once. Adult female flies were gently placed inside the central arm of the Y-tube from a glass vial and observed for 6 min. Flies remaining unresponsive for 5 min were labelled as no-choice. For each responsive female the latent period (i.e. time spent inside the arena before making a choice) and the choice (i.e. the odour source selected) were recorded. The choice was recorded when a responsive fly went inside one of the spherical trap chambers at the end of the Y-tube arms and remained there for at least 30 seconds.

### Preferences of *B. oleae* females toward olive fruits

For the three tested olive varieties (cv. Ottobratica, Roggianella and Sinopolese), the preferences of *B. oleae* females toward fruits with different maturation degree and *B. oleae*-infestation status was evaluated. Four treatments were evaluated for each cultivar: LI-Green; LI-Black; HI-Green; HI-Black. To assess if the different levels of *B. oleae*-infestation, along with the maturation degree of the fruit, could affect the orientation of *B. oleae* females toward olive drupes, the following odour sources were compared in Y-tube trials:

(i) LI-Green vs LI-Black (4 drupes; ca 10 g);
(ii) HI-Green vs HI-Black (4 drupes; ca 10 g);
(iii) LI-Green vs HI-Green (4 drupes; ca 10 g);
(iv) LI-Black vs HI-Black (4 drupes; ca 10 g).

The above listed comparisons were provided for the three tested cultivars separately. The odorous sources consisted in four olive fruits, which were replaced with four new fruits every three replicates. Thirty responsive adult females were tested individually for each comparison.

### Effect of *B. oleae* infestation and fruit maturation on VOC emissions

For the three tested olive varieties (cv. Ottobratica, Roggianella and Sinopolese), the VOC emissions of the four different treatments used in the bioassays were evaluated. For all treatments, 4 olive fruits (ca. 10 g) were inserted into a 30-mL hermetic glass vial and allowed to equilibrate for 30 min. Sampling was accomplished in an air-conditioned room (22 ± 1 °C) to guarantee a stable temperature. Three replicates (i.e. each containing 4 olive fruits) were performed for all treatments. To sample the headspace of the olives a Supelco® (Bellefonte, PA, USA) SPME device coated with polydimethylsiloxane (PDMS, 100 μm) was used. SPME sampling was performed using the same new fiber, preconditioned according to the manufacturer instructions. After the equilibration time, the fiber was exposed to the headspace for 30 min. Once sampling was finished, the fiber was withdrawn into the needle and transferred to the injection port of the GC-MS system. The fiber was desorbed for 5 minutes. All the SPME sampling and desorption conditions were identical for all the samples. Blanks were performed before first SPME extraction and randomly repeated during each series. Quantitative comparisons of relative peaks areas were performed between the same identified chemicals in different samples.

GC-MS analyses were performed with a Thermo Fisher TRACE 1300 gas chromatograph equipped with a DB-5 capillary column (30 m x 0.25 mm; coating thickness = 0.25 μm) and a Thermo Fisher ISQ LT ion trap mass detector (emission current: 10 microamps; count threshold: 1 count; multiplier offset: 0 volts; scan time: 1.00 second; prescan ionization time: 100 microseconds; scan mass range: 30–300 m/z; ionization mode: EI). The following analytical conditions were used: injector and transfer line temperature at 250 and 240 °C, respectively; oven temperature programmed from 60 to 240 °C at 3 °C min^−1^; carrier gas, helium at 1 mL min^−1^; splitless injection. Molecule identification was based on comparison of their linear retention indices (LRI) relative to the series of n-hydrocarbons, comparing the retention times (RT) with those of pure chemicals, and on computer matching against commercial (NIST 98 and ADAMS) and homemade library mass spectra built from pure substances, components of known oils and MS literature data^45–47^.

### Behavioural responses of *B. oleae* females toward selected synthetic VOCs

All pure chemicals used for bioassays [n-hexane (≥96%), *β*-myrcene (≥98%), limonene (≥99%), *β*-ocimene (≥98%)] were purchased from Sigma Aldrich (Munich, Germany). These volatiles were selected because, according to statistics, their emissions were significantly altered by infestation status in all the tested cultivars.

Two different concentrations of each synthetic HIPV (1 and 10 μg/μL) were dissolved in n-hexane and offered to adult *B. oleae* females in Y-tube bioassays. VOC concentrations used in the bioassays were chosen according to previous results on the EAG and GC-EAD responses of *B. oleae* toward pure chemicals and extracts^44,48^. Electro-antennographical analyses determine the perception of different stimuli directly by the antennal sensilla, providing biological information on insect perceptions and facilitating the identification of a low and a high concentration to be used in the olfactometer. For each trial, 5 μl of VOC-solution was applied to a filter paper (1.5 × 1.5 cm Whatman no. 1) and, after solvent evaporation (ca. 20 s), it was inserted into a Drechsel bottle and connected to an arm of the Y-tube olfactometer. Equally, the other Y-tube arm was connected to a Drechsel bottle containing a similar filter paper treated with 5 μl of pure n-hexane as control. Following trials were performed comparing in Y-tube 5 μl of VOC-solution at 1 μg/μL and 5 μl of the same VOC at 10 μg/μL.The filter papers were renewed every three replicates. A total of thirty adult female flies were tested for every trial.

### Data analyses

All the described statistical analyses were achieved using the software JMP 11^®^. Concerning the bioassays, for every trial, a likelihood chi-square test with Yates correction (with *α* = 0.05) was used to compare the proportion of flies choosing a given cue. The latent periods were processed by a non-parametric model, the Mann-Whitney U test, assuming no normality of data by Shapiro-Wilk test.

Before statistical analysis of VOC emissions, the area integration report of every identified compound and chemical class was transformed into Log values. The normal distribution of selected data was also checked using Shapiro–Wilk test (*P* > 0.05). For every tested cultivar, a GLM with two fixed factors (infestation status and maturation) [yj = *μ* + Ij + Mj + (Ij × Mj) + ej, in which yj is the observation, *μ* is the overall mean, Ij the fruit infestation status (j = 1–2), Mj the maturation status (j = 1–2), Ij × Mj the interaction infestation × maturation status, and ej the residual error] was performed, to evaluate possible alteration in volatile emissions between the different treatments. False discovery rate (FDR; Benjamini and Hochberg^49^) was assessed to control the experiment-wide error rate provided by the large number of comparisons.

To investigate the different sources of VOC variability, which can be attributable to genetic differences (i.e. varieties), as well as to common factors (i.e. infestation status and maturation), Principal Component Analysis (PCA) was achieved on transformed values of each VOC. To reveal clusters related to the original variables (variety, maturation and infestation status), two-dimensional score plots were produced. Next, a Multi-Factorial Analysis (MFA) was performed using a principal component procedure and a VARIMAX orthogonal rotation technique. The rotated factors with an eigenvector of at least ± 0.5 were considered to label each factor according to the involved source of variability. Furthermore, scores of common factors were calculated as described by Macciotta *et al*.^50^ and analysed using a GLM with three fixed factors (variety, maturation and infestation status). Step-wise discriminant analyses were also completed to find a set of variables (with R^2^ > 0.1) highly representative of fixed factors (i.e. infestation status and maturation degree). The ratio (Wilks lambda) between the generalized within-category dispersion and the total dispersion was taken into account^51^.

## Supplementary information

Supplementary Information.

## Data Availability

The datasets generated during and/or analysed during the current study are available from the corresponding author on reasonable request.
