# Supplementary material for: Olive fruit volatiles route intraspecific interactions and chemotaxis in Bactrocera oleae (Rossi) (Diptera: Tephritidae) females
Source: Sci Rep. 2020 Feb 3;10:1666. doi: 10.1038/s41598-020-58379-8 (PMC6997409; doi:10.1038/s41598-020-58379-8)

Olive fruit volatiles route intraspecific interactions and chemotaxis in *Bactrocera oleae* (Rossi) (Diptera: Tephritidae) females

Giulia Giunti, Orlando Campolo [@], Francesca Laudani, Giuseppe Massimo Algeri, Vincenzo Palmeri

Department of Agriculture, University "Mediterranea" of Reggio Calabria, Loc. Feo di Vito, 89122 Reggio Calabria, Italy

[@] Corresponding author.

Address: Loc. Feo di Vito, 89122 Reggio Calabria, Italy

tel: +39 0965 1694266; e-mail address: orlando.campolo@unirc.it

Supplementary Table S1 Mean (±SE) latent period (s) of *Bactrocera oleae* females on different odour sources in two-choice bioassays conducted in Y-tube olfactometer. Olive fruits with different maturation degree and infestation status were compared. Different doses (1 or 10 µg/µL) of VOCs were tested versus pure hexane (blank). Thirty females were tested for every trial.

**Cultivar**	**Stimulus 1**	**Latent Period 1 (Mean ± SE)**	**Stimulus 2**	**Latent Period 2 (Mean ± SE)**
Ottobratica	Green HI	121.67 ± 59.32	Green LI	127.24 ± 26.02
	Black HI	139.2 ± 53.52	Black LI	99.87 ± 23.32
	Green HI	109.89 ± 37.44	Black HI	100.09 ± 30.41
	Green LI	104.5 ± 27.85	Black LI	91.25 ± 33.48
Sinopolese	Green HI	181 ± 82.53	Green LI	131 ± 23.76
	Black HI	115.75 ± 62.33	Black LI	95.56 ± 23.59
	Green HI	136.5 ± 31.45	Black HI	114 ± 33.29
	Green LI	139.87 ± 29.24	Black LI	101.6 ± 40.6
Roggianella	Green HI	180.4 ± 42.74	Green LI	146.27 ± 29.23
	Black HI	158 ± 58.93	Black LI	122.67 ± 24.79
	Green HI	133.2 ± 31.49	Black HI	127.7 ± 26.93
	Green LI	132.75 ± 27.34	Black LI	138.88 ± 42.39
**Chemical**	**Stimulus 1**	**Latent Period 1 (Mean ± SE)**	**Stimulus 2**	**Latent Period 2 (Mean ± SE)**
β-ocimene	1 µg/µL	177.46 ± 31	Blank	148.47 ± 25.42
	10 µg/µL	87.88 ± 34.94	Blank	92.32 ± 20.92
	1 µg/µL	138.24 ± 23.19	10 µg/µL	126.89 ± 24.07
β-myrcene	1 µg/µL	125.33 ± 21.88	Blank	139.13 ± 29.6
	10 µg/µL	82.35 ± 20.23	Blank	101.46 ± 29.97
	1 µg/µL	140.21 ± 30.7	10 µg/µL	127.75 ± 28.02
limonene	1 µg/µL	130.33 ± 28.45	Blank	111.47 ± 25.77
	10 µg/µL	116.88 ± 32.98	Blank	98.5 ± 19.93
	1 µg/µL	118.74 ± 22.05	10 µg/µL	128 ± 24.12

Supplementary Table S 2 Volatiles emitted by olive fruits from cultivar Ottobratica according to *Bactrocera oleae* infestation and maturation degree. Peak area values are provided as mean ± standard error of three replicates. Statistical values (F ratio and *P* value) are specified only for single chemicals and chemical classes showing significant differences. Different letters represent significant differences between the values of the same row (Tukey's HSD).

LRI= Linear Retention Index; HI= High Infestation; LI= Low Infestation

Chemical classes	LRI	Compound	Peak Area (Mean ± SE)				$F_{3,8}$	*P* value
			HI-Black	HI-Green	LI-Black	LI-Green		
**Hydrocarbons**	1100	*n*-Undecane	4604.15 ± 1143.34	4359.30 ± 4354.30	5896.04 ± 1371.75	4972.09 ± 1387.82		
	1200	*n*-Dodecane	20981.00 ± 2608.61	15749.62 ± 1729.13	14428.40 ± 2692.88	15457.70 ± 2608.69		
	1215	1-Dodecene	0.00 ± 0.00	0.00 ± 0.00	3527.48 ± 1766.85	0.00 ± 0.00		
	1300	*n*-Tridecane	11697.61 ± 3209.30	6191.40 ± 147.84	12140.22 ± 1453.95	12620.77 ± 1634.35		
	1313	(6*Z*)-6-Tridecene	tr	0.00 ± 0.00	0.00 ± 0.00	1423.37 ± 713.26		
	1370	3-Methyltridecane	tr<br>b	tr<br>b	3269.78 ± 725.95<br>a	2611.06 ± 543.43<br>a	5.79	0.021
	1391	(4*E*)-4-Tetradecene	1653.45 ± 1643.45<br>ab	0.00 ± 0.00<br>b	4206.19 ± 897.47<br>a	2627.61 ± 476.28<br>a	62.06	<0.0001
	1400	*n*-Tetradecane	4619.43 ± 807.50	6066.85 ± 135.17	11408.40 ± 3058.50	8662.81 ± 1876.24		
	1500	*n*-Pentadecane	1942.24 ± 142.85<br>b	tr<br>c	5320.82 ± 680.79<br>a	5221.68 ± 1482.50<br>a	318.04	<0.0001
	1600	*n*-Hexadecane	2193.35 ± 1110.08	tr	5845.85 ± 1003.26	3682.60 ± 417.79		
	1700	*n*-Heptadecane	544.86 ± 534.85<br>b	tr<br>b	5141.92 ± 1641.72<br>a	5968.99 ± 1036.80<br>a	13.65	0.002
	1800	*n*-Octadecane	1835.04 ± 1065.46	tr	2104.09 ± 1209.18	0.00 ± 0.00		
		**Σ Hydrocarbons**	**51388.23 ± 5846.22<br>ab**	**34741.92 ± 2568.85<br>b**	**73289.19 ± 12934.03<br>a**	**63248.69 ± 6269.77<br>a**	**5.92**	**0.020**

Chemical classes	LRI	Compound	Peak Area (Mean ± SE)				$F_{3,8}$	*P* value
			HI-Black	HI-Green	LI-Black	LI-Green		
**Aldehydes**	1005	Nonanal	87784.11 ± 8380.94 a	86440.38 ± 1438.04 a	34190.84 ± 1715.95 b	34697.28 ± 1555.06 b	78.01	<0.0001
	1206	Decanal	89309.39 ± 25817.00 b	196239.66 ± 11739.94 a	40222.03 ± 2773.87 c	28548.25 ± 1845.81 c	26.75	<0.001
	1297	10-Undecenal	1436.59 ± 743.66 a	6328.83 ± 1017.95 a	0.00 ± 0.00 b	0.00 ± 0.00 b	9.9101	0.005
	1308	Undecanal	6568.15 ± 2340.27	15000.41 ± 1963.09	3891.43 ± 746.15	8230.23 ± 5837.38		
	1409	Dodecanal	5250.31 ± 1516.45 ab	12040.47 ± 503.45 a	2700.01 ± 510.10 b	3763.55 ± 422.36 b	9.55	0.005
	1447	11-Dodecenal	tr ab	0.00 ± 0.00 a	4230.74 ± 1640.96 b	tr ab	4.71	0.036
	1613	Tetradecanal	1332.84 ± 1327.84 ab	1446.17 ± 724.54 a	0.00 ± 0.00 b	0.00 ± 0.00 b	7.5746	0.010
		**Σ Aldehydes**	**191688.04 ± 35983.82 b**	**317495.92 ± 12797.30 a**	**85235.05 ± 3643.51 c**	**75689.05 ± 7809.57 c**	**33.09**	**<0.0001**
**Alcohols**	1168	(2*E*)-2-Nonen-1-ol	613.77 ± 603.77 ab	tr b	2313.51 ± 206.50 a	2651.29 ± 702.56 a	9.64	0.005
	1174	1-Nonanol	16454.42 ± 2941.86	2301.95 ± 1174.48	9713.31 ± 1152.94	7639.87 ± 863.50		
	1241	1-Decanol	0.00 ± 0.00	0.00 ± 0.00	tr	tr		
	1278	(2*E*)-2-Decen-1-ol	3480.28 ± 1745.81	8173.26 ± 969.91	3199.35 ± 294.42	1761.15 ± 434.21		
	1476	1-Dodecanol	1969.23 ± 1046.85	0.00 ± 0.00	0.00 ± 0.00	0.00 ± 0.00		
	1691	1-Tetradecanol	412992.28 ± 88754.75 a	819197.28 ± 248458.71 a	125771.39 ± 24334.39 b	96549.54 ± 21186.73 b	14.53	0.001
		**Σ Alcohols**	**435509.98 ± 84505.50 a**	**829682.49 ± 246935.41 a**	**141549.72 ± 24978.58 b**	**109152.09 ± 21497.18 b**	**15.22**	**0.001**
**Ketones**	1118	2-Methyl-6-methylene-1,7-octadien-3-one	3141.28 ± 2101.54 c	221055.20 ± 14728.70 a	4008.86 ± 1102.01 c	9143.32 ± 2532.45 b	4.52	0.039

Chemical classes	LRI	Compound	Peak Area (Mean ± SE)				$F_{3,8}$	*P* value
			HI-Black	HI-Green	LI-Black	LI-Green		
		**Σ Ketones**	**3141.28 ± 2101.54 c**	**221055.20 ± 14728.70 a**	**4008.86 ± 1102.01 c**	**9143.32 ± 2532.45 b**	**4.52**	**0.039**
**Esters**	1010	(4*E*)-4-Hexen-1-yl acetate	tr	15937.58 ± 8026.75	21453.14 ± 397.55	21583.70 ± 2638.67		
	1015	Hexyl acetate	tr	0.00 ± 0.00	31437.16 ± 3081.50	0.00 ± 0.00	23.55	<0.001
		**Σ Esters**	**tr**	**15937.58 ± 8026.75**	**52890.30 ± 7006.08**	**21583.70 ± 2638.67**		
**Monoterpenes hydrocarbons**	992	*β*-myrcene	10588.85 ± 2960.57 a	4310.02 ± 1784.69 ab	7508.02 ± 1412.97 a	tr b	64.02	<0.0001
	1017	*α*-terpinene	11385.55 ± 1536.28 a	tr b	0.00 ± 0.00 c	0.00 ± 0.00 c	19697.45	<0.0001
	1025	*o*-cymene	36103.57 ± 3840.67 a	1490.98 ± 1480.98 b	0.00 ± 0.00 c	0.00 ± 0.00 c	94.91	<0.0001
	1029	Limonene	1039345.68 ± 141906.88 a	651160.04 ± 172917.62 a	3749.90 ± 331.66 b	5523.38 ± 716.40 b	317.41	<0.0001
	1048	(*E*)-*β*-ocimene	52462.32 ± 1683.64 b	1469327.74 ± 237536.18 a	5113.16 ± 708.36 c	3035.84 ± 276.96 c	578.61	<0.0001
	1059	*γ*-terpinene	450621.55 ± 66524.07 a	304819.43 ± 88057.13 a	0.00 ± 0.00 b	0.00 ± 0.00 b	6444.92	<0.0001
	1089	*α*-terpinolene	1849.05 ± 3443.06 a	tr ab	0.00 ± 0.00 b	tr ab	7.17	0.012
	1131	*trans*-alloocimene	tr	tr	0.00 ± 0.00	0.00 ± 0.00		
		**Σ Monoterpenes hydrocarbons**	**1619007.56 ± 212702.62 a**	**2426838.20 ± 181654.70 a**	**16371.08 ± 1524.16 b**	**15305.02 ± 273.56 b**	**901.06**	**<0.0001**
**Monoterpenes oxigenated**	1178	4-terpineol	0.00 ± 0.00	tr	0.0 ± 0.00	0.00 ± 0.0		
	1192	*α*-terpineol	12923.90 ± 3405.91	7668.36 ± 4938.45	6652.96 ± 2425.89	4515.58 ± 1473.99		
	1373	Limonene diepoxide	4756.28 ± 3340.58	8776.76 ± 1238.37	1435.36 ± 779.18	587.30 ± 298.02		

Chemical classes	LRI	Compound	Peak Area (Mean ± SE)				$F_{3,8}$	***P* value**
			**HI-Black**	**HI-Green**	**LI-Black**	**LI-Green**		
		**Σ Monoterpenes oxigenated**	**17680.18 ± 5305.72**	**16455.12 ± 4997.53**	**8088.32 ± 2885.66**	**5102.88 ± 1637.66**		
**Sesquiterpenes hydrocarbons**	1344	*α*-longipinene	1234.37 ± 1224.37<br>a	tr<br>ab	tr<br>ab	0.00 ± 0.00<br>b	4.26	0.045
	1365	Cyclosativene	1158.79 ± 577.79<br>b	23635.07 ± 7560.55<br>a	3179.64 ± 481.33<br>ab	3595.89 ± 902.36<br>ab	3.67	0.063
	1368	Longicyclene	tr	1241.80 ± 1231.80	310.00 ± 300.00	469.09 ± 254.93		
	1376	*α*-copaene	5831.36 ± 638.17<br>b	182910.70 ± 30772.84<br>a	5884.76 ± 658.27<br>b	7172.78 ± 491.83<br>b	174.15	<0.0001
	1380	Isolongifolene	tr<br>ab	1256.93 ± 1246.93<br>a	0.00 ± 0.00<br>b	0.00 ± 0.00<br>b	9.22	0.006
	1420	*β*-caryophyllene	15659.97 ± 12533.96	34688.74 ± 8666.45	793.24 ± 440.54	2151.89 ± 384.12		
	1429	*β*-copaene	0.00 ± 0.00<br>b	4350.37 ± 2365.76<br>a	0.00 ± 0.00<br>b	8938.89 ± 514.29<br>a	42.84	<0.0001
	1437	(*Z*)-*α*-bergamotene	1822.68 ± 913.51<br>a	6404.52 ± 1615.74<br>a	0.00 ± 0.00<br>b	0.00 ± 0.00<br>b	103.65	<0.0001
	1486	*β*-selinene	tr	2585.56 ± 2575.56	tr	1170.33 ± 225.42		
	1489	Valencene	7495.14 ± 1965.04<br>b	25429.15 ± 4881.14<br>a	1840.86 ± 365.81<br>c	2080.94 ± 83.47<br>c	37.40	<0.0001
	1496	*α*-selinene	tr	3100.98 ± 1553.57	tr	975.78 ± 592.00		
	1502	*α*-muurulene	0.00 ± 0.00<br>c	12128.66 ± 3287.58<br>a	0.00 ± 0.00<br>c	516.42 ± 271.82<br>b	170.46	<0.0001
	1510	(*E*,*E*)-*α*-farnesene	30637.42 ± 11740.36<br>b	119608.63 ± 2536.71<br>a	2890.33 ± 602.11<br>c	3154.73 ± 649.57<br>c	42.06	<0.0001
		**Σ Sesquiterpenes hydrocarbons**	**63863.06 ± 5661.26<br>b**	**417351.11 ± 56523.24<br>a**	**15517.37 ± 344.82<br>c**	**22181.73 ± 573.96<br>c**	**278.72**	**<0.0001**
**Sesquiterpenes oxigenated**	1532	Dihydro-β-agarofuran	0.00 ± 0.00<br>b	19015.81 ± 3093.39<br>a	0.00 ± 0.00<br>b	0.00 ± 0.00<br>b	11051.<br>14	<0.0001

Chemical classes	LRI	Compound	Peak Area (Mean ± SE)				$F_{3,8}$	*P* value
			HI-Black	HI-Green	LI-Black	LI-Green		
	1559	Longipinanol	0.00 ± 0.00 b	6877.64 ± 4850.69 a	0.00 ± 0.00 b	0.00 ± 0.00 b	884.86	<0.0001
	1619	Epi-cedrol	0.00 ± 0.00	2951.97 ± 2941.97	tr	tr		
	1664	*α*-bisabolol	3358.09 ± 1937.11	4470.52 ± 2241.15	3610.14 ± 526.71	1913.98 ± 483.99		
	1842	Farnesol acetate	4672.59 ± 814.97	29142.90 ± 2802.37	3656.83 ± 1995.96	4476.06 ± 792.80		
		**Σ Sesquiterpenes oxigenated**	**8219.68 ± 2704.33 b**	**64494.63 ± 7621.10 a**	**7629.17 ± 1889.39 b**	**6561.37 ± 1060.46 b**	**11.76**	**0.003**
**Apocarotenoids**	1455	Geranylacetone	12129.28 ± 2390.29 b	116377.24 ± 3353.26 a	16503.68 ± 6100.14 b	11527.68 ± 2109.59 b	23.95	<0.001
		**Σ Apocarotenoids**	**12129.28 ± 2390.29 b**	**116377.24 ± 3353.26 a**	**16503.68 ± 6100.14 b**	**11527.68 ± 2109.59 b**	**23.95**	**<0.001**

Supplementary Table S3 Volatiles emitted by olive fruits from cultivar Sinopolese according to *Bactrocera oleae* infestation and maturation degree. Peak area values are provided as mean ± standard error of three replicates. Statistical values (F ratio and *P* value) are specified only for single chemicals and chemical classes showing significant differences. Different letters represent significant differences between the values of the same row (Tukey's HSD).

LRI= Linear Retention Index; HI= High Infestation; LI= Low Infestation

**Chemical classes**	**LRI**	**Compound**	**Peak Area (Mean SE)**				**$F_{3,8}$**	***P* value**
			**HI-Black**	**HI-Green**	**LI-Black**	**LI-Green**		
**Hydrocarbons**	1100	*n*-Undecane	3116.18 ± 1029.74 ab	10876.95 ± 2450.60 a	0.00 ± 0.00 c	1280.05 ± 650.31 b	90.14	<0.0001
	1200	*n*-Dodecane	16056.39 ± 1141.69 ab	47896.10 ± 4887.79 a	6689.76 ± 443.94 c	13194.97 ± 4634.88 bc	10.51	0.004
	1300	*n*-Tridecane	11658.18 ± 3441.81 ab	40156.96 ± 4721.34 a	8471.46 ± 778.94 b	7353.86 ± 2867.07 b	6.58	0.015
	1370	3-Methyltridecane	1677.49 ± 118.89	2009.92 ± 1008.64	838.32 ± 478.73	4763.11 ± 2473.1		
	1391	(4*E*)-4-Tetradecene	3239.07 ± 979.12	tr	tr	2254.99 ± 1217.48		
	1400	*n*-Tetradecane	5942.58 ± 285.39	6289.08 ± 941.19	5123.33 ± 1181.37	9355.74 ± 1842.62		
	1500	*n*-Pentadecane	tr	7952.96 ± 3837.69	467.45 ± 280.19	1739.03 ± 935.09		
	1600	*n*-Hexadecane	1909.92 ± 950.13	19931.26 ± 7099.65	5300.73 ± 1747.91	5008.99 ± 1413.26		
	1700	*n*-Heptadecane	tr	16395.41 ± 6740.17	tr	2562.71 ± 1283.00		
	1800	*n*-Octadecane	tr b	7492.15 ± 2612.77 a	1775.82 ± 1765.82 ab	2782.32 ± 577.19 a	7.89	0.009
	1900	*n*-Nonadecane	0.00 ± 0.00 b	9418.92 ± 5918.85 a	tr ab	tr ab	7.25	0.011
	2000	*n*-Eicosane	0.00 ± 0.00	1040.90 ± 522.79	0.00 ± 0.00	0 ± 0		
		**Σ Hydrocarbons**	**44105,05 ± 4369.05 b**	**169467.28 ± 22539.66 a**	**29341.73 ± 6033.87 c**	**50302.42 ± 16527.83 bc**	**8.91**	**0.006**

Chemical classes	LRI	Compound	Peak Area (Mean SE)				$F_{3,8}$	*P* value
			**HI-Black**	**HI-Green**	**LI-Black**	**LI-Green**		
**Aldehydes**	1005	Nonanal	54416,26 ± 4825.50 a	62901.4 ± 3646.79 a	29096.56 ± 732.40 b	31844.69 ± 9820.51 b	4.93	0.032
	1206	Decanal	69262.26 ± 5825.69	90200.37 ± 21740.86	73686.25 ± 1286.29	66397.17 ± 19449.94		
	1308	Undecanal	7193.11 ± 902.98	8399.66 ± 1527.77	5948.45 ± 313.34	5260.70 ± 1803.37		
	1409	Dodecanal	3461.22 ± 387.63	5379.43 ± 741.18	5596.46 ± 1986.00	3431.74 ± 45.27		
	1447	11-Dodecenal	1918.67 ± 966.36	tr	tr	tr		
		**Σ Aldehydes**	**136251.52 ± 3034.80**	**166890.86 ± 21872.87**	**114337.72 ± 1900.83**	**106937.64 ± 30344.21**		
**Alcohols**	1174	1-Nonanol	4389.96 ± 1429.68	4535.38 ± 911.94	3774.25 ± 347.05	4042.96 ± 1774.00		
	1241	1-Decanol	tr ab	3212.38 ± 280.04 a	0.00 ± 0.00 b	2038.51 ± 1147.52 ab	5.02	0.030
	1278	(2*E*)-2-Decen-1-ol	2659.81 ± 365.12	5712.94 ± 615.35	3990.96 ± 642.85	4492.15 ± 1394.12		
	1476	1-Dodecanol	tr	2298.03 ± 1171.54	tr	1001.34 ± 996.34		
	1691	1-Tetradecanol	947975.35 ± 212196.81	296786.6 ± 122104.69	641814.88 ± 298307.61	812159.54 ± 313113.35		
		**Σ Alcohols**	**955035.12 ± 210616.91**	**312545.32 ± 123149.51**	**650259.35 ± 298250.18**	**823734.49 ± 315597.5**		
**Ketones**	1118	2-Methyl-6-methylene-1,7-octadien-3-one	2986.62 ± 600.45 b	29689.54 ± 14619.09 a	3414.16 ± 101.33 b	5197.22 ± 1218.46 ab	10.56	0.004
		**Σ Ketones**	**2986.62 ± 600.45 b**	**29689.54 ± 14619.09 a**	**3414.16 ± 101.33 b**	**5197.22 ± 1218.46 ab**	**10.56**	**0.004**
**Esters**	1110	(4*E*)-4-Hexen-1-yl acetate	tr	0.00 ± 0.00	5159.30 ± 3011.26	tr		
	1015	Hexyl acetate	12440.35 ± 12440.34 a	0.00 ± 0.00 b	10036.36 ± 5698.84 a	0.00 ± 0.00 b	4.86	0.033
		**Σ Esters**	**17565.37 ± 17565.37 ab**	**0.00 ± 0.00 b**	**15195.66 ± 4798.23 a**	**tr ab**	**4.45**	**0.041**

Chemical classes	LRI	Compound	Peak Area (Mean SE)				F $_{3,8}$	*P* value
			HI-Black	HI-Green	LI-Black	LI-Green		
**Monoterpenes hydrocarbons**	992	*β*-myrcene	15490.98 ± 10376.10 a	9848.48 ± 281.56 a	tr b	638.36 ± 326.78 b	3.98	0.050
	1017	*α*-terpinene	5172.09 ± 3169.61 a	17865.66 ± 3045.29 a	0.00 ± 0.00 b	0.00 ± 0.00 b	9.69	0.005
	1025	*o*-cymene	8889.86 ± 4764.18 b	67234.25 ± 16066.17 a	0.00 ± 0.00 c	0.00 ± 0.00 c	10.22	0.004
	1029	Limonene	225922.97 ± 56472.13 ab	1341753.76 ± 260585.61 a	6012.69 ± 656.01 ab	692.89 ± 346.97 b	4.20	0.047
	1048	(*E*)-*β*-ocimene	12808.75 ± 1487.49 a	150666.02 ± 26338.10 a	4001.76 ± 3856.82 b	tr b	4.22	0.046
	1059	*γ*-terpinene	100545.32 ± 28633.02 b	674363.00 ± 119257.09 a	0.00 ± 0.00 c	0.00 ± 0.00 c	5637.88	<0.0001
	1089	*α*-terpinolene	2481.75 ± 1625.59 b	34838.48 ± 368.82 a	0.00 ± 0.00 c	0.00 ± 0.00 c	11.06	0.003
	1131	*trans*-alloocimene	0.00 ± 0.00 b	4013.64 ± 494.85 a	0.00 ± 0.00 b	tr ab	11.95	0.003
		**Σ Monoterpenes hydrocarbons**	**371311.73 ± 93278.32 b**	**2300583.30 ± 417083.07 a**	**10017.78 ± 3385.63 c**	**2082.97 ± 370.05 d**	**178.38**	**<0.0001**
**Monoterpenes oxigenated**	1178	4-terpineol	tr b	3164.49 ± 774.65 a	0.00 ± 0.00 b	0.00 ± 0.00 b	18.02	<0.001
	1192	*α*-terpineol	13804.81 ± 5986.90	12109.75 ± 1558.90	8742.66 ± 7125.27	4487.41 ± 2306.45		
	1373	Limonene diepoxide	3355.00 ± 237.78	6368.03 ± 379.61	2229.43 ± 1468.53	1716.91 ± 328.33		
		**Σ Monoterpenes oxigenated**	**17159.81 ± 6225.28**	**21642.27 ± 2528.71**	**10972.09 ± 8593.80**	**6204.32 ± 2634.78**		
**Sesquiterpenes hydrocarbons**	1365	Cyclosativene	0.00 ± 0.00	0.00 ± 0.00	0.00 ± 0.00	7920.74 ± 4278.10		
	1368	Longicyclene	0.00 ± 0.00	0.00 ± 0.00	0.00 ± 0.00	4377.53 ± 2799.15		
	1376	*α*-copaene	tr	tr	1350.88 ± 738.48	78046.82 ± 44599.95		

Chemical classes	LRI	Compound	Peak Area (Mean SE)				$F_{3,8}$	*P* value
			HI-Black	HI-Green	LI-Black	LI-Green		
	1380	isolongifolene	3453.03 ± 2152.02	11243.34 ± 2590.58	tr	tr		
	1420	*β*-caryophyllene	6295.47 ± 1864.83	10109.65 ± 3374.74	2322.02 ± 436.49	3159.25 ± 1105.90		
	1429	*β*-copaene	tr	3006.35 ± 1540.02	tr	2506.56 ± 643.59		
	1437	(*Z*)-*α*-bergamotene	2561.19 ± 481.41 ab	6952.70 ± 1796.11 a	2168.15 ± 539.05 ab	1923.58 ± 990.55 b	4.12	0.048
	1458	(*E*)-*β*-farnesene	tr b	2556.78 ± 1483.04 ab	tr b	6667.47 ± 3335.23 a	3.98	0.049
	1486	*β*-selinene	1694.06 ± 856.63	3152.58 ± 1588.28	tr	tr		
	1489	Valencene	4287.75 ± 435.43 ab	16429.94 ± 2349.91 a	3203.43 ± 1605.41 b	3438.90 ± 1342.81 b	3.99	0.049
	1496	*α*-selinene	tr	2319.81 ± 1436.06	tr	tr		
	1502	*α*-muurulene	0.00 ± 0.00 b	tr ab	0.00 ± 0.00 b	2951.59 ± 1599.16 a	5.24	0.027
	1510	(*E,E*)-*α*-farnesene	5936.50 ± 535.25 b	68431.11 ± 34256.43 a	22114.83 ± 13423.47 ab	7844.26 ± 2997.04 ab	4.77	0.034
		**Σ Sesquiterpenes hydrocarbons**	**24868.55 ± 1169.07**	**124218.92 ± 48296.35**	**32115.06 ± 15051.60**	**112609.08 ± 57391.96**		
**Sesquiterpenes oxigenated**	1531	liguloxide	0.00 ± 0.00	0.00 ± 0.00	0.00 ± 0.00	4883.00 ± 2753.03		
	1619	Epi-cedrol	tr	tr	tr	tr		
	1664	*α*-bisabolol	6990.76 ± 4968.57	1946.62 ± 1112.91	3875.49 ± 1485.48	147.38 ± 104.13		
	1842	Farnesol acetate	7834.02 ± 1989.22	7781.76 ± 2622.58	4346.36 ± 1139.47	6000.64 ± 1938.65		
		**Σ Sesquiterpenes oxigenated**	**14824.78 ± 6662.65**	**9728.38 ± 3869.37**	**8221.85 ± 3064.87**	**11031.02 ± 3808.49**		
**Apocarotenoids**	1455	Geranylacetone	25276.88 ± 4248.65	44238.22 ± 23680.92	12833.58 ± 4390.64	21949.28 ± 6724.60		

Chemical classes	LRI	Compound	Peak Area (Mean SE)				$F_{3,8}$	*P* value
			HI-Black	HI-Green	LI-Black	LI-Green		
		**Σ Apocarotenoids**	**25276.88 ± 4248.65**	**44238.22 ± 23680.92**	**12833.58 ± 4390.64**	**7920.74 ± 4278.1**		

Supplementary Table S4 Volatiles emitted by olive fruits from cultivar Roggianella according to *Bactrocera oleae* infestation and maturation degree. Peak area values are provided as mean ± standard error of three replicates. Statistical values (F ratio and *P* value) are specified only for single chemicals and chemical classes showing significant differences. Different letters represent significant differences between the values of the same row (Tukey's HSD).

LRI= Linear Retention Index; HI= High Infestation; LI= Low Infestation

Chemical classes	LRI	Compound	Peak Area (Mean ± SE)				$F_{3,8}$	*P* value
			HI-Black	HI-Green	LI-Black	LI-Green		
**Hydrocarbons**	1100	*n*-Undecane	5956.43 ± 1186.74 a	tr b	2974.44 ± 251.11 a	tr b	4.63	0.037
	1200	*n*-Dodecane	15249.95 ± 2001.36 a	5825.20 ± 1376.77 b	7199.36 ± 277.40 ab	5748.67 ± 1030.65 b	7.17	0.012
	1300	*n*-Tridecane	10371.94 ± 3487.28	10140.10 ± 935.58	7629.88 ± 1277.24	10129.29 ± 2712.85		
	1370	3-Methyltridecane	3953.45 ± 660.78	1194.28 ± 802.57	2047.86 ± 208.46	tr		
	1391	(4*E*)-4-Tetradecene	6125.67 ± 1279.04	tr	1936.70 ± 1077.50	tr		
	1400	*n*-Tetradecane	6772.04 ± 798.57 a	3936.71 ± 335.00 bc	5001.65 ± 285.59 ab	2685.00 ± 238.03 c	19.09	<0.001
	1500	*n*-Pentadecane	4980.70 ± 1092.06	3843.66 ± 1258.62	3881.18 ± 524.05	1614.03 ± 406.81		
	1600	*n*-Hexadecane	5518.39 ± 830.31	5129.08 ± 1677.28	5653.05 ± 1920.91	4202.83 ± 1113.99		
	1700	*n*-Heptadecane	5425.77 ± 407.57	3744.49 ± 1915.83	5219.62 ± 797.15	6670.22 ± 3613.23		
	1800	*n*-Octadecane	2334.58 ± 844.88	2423.91 ± 527.62	2499.82 ± 267.01	2221.10 ± 463.62		
		**Σ Hydrocarbons**	**66688.91 ± 9467.17 a**	**36771.08 ± 4940.48 b**	**44010.56 ± 5037.24 ab**	**33926.31 ± 2886.83 b**	**6.10**	**0.018**
**Aldehydes**	1105	Nonanal	35999.84 ± 3288.91	27862.55 ± 4716.20	27521.72 ± 691.72	19993.33 ± 5815.37		
	1206	Decanal	96151.46 ± 12343.56	121067.09 ± 11789.14	139535.78 ± 14610.75	109588.01 ± 24373.44		

Chemical classes	LRI	Compound	Peak Area (Mean ± SE)				$F_{3,8}$	*P* value
			**HI-Black**	**HI-Green**	**LI-Black**	**LI-Green**		
	1308	Undecanal	3839.03 ± 412.49	7933.99 ± 1105.36	6465.57 ± 934.23	7726.08 ± 2095.60		
	1409	Dodecanal	4569.44 ± 720.81	4532.74 ± 491.69	4837.01 ± 418.49	5451.50 ± 666.20		
	1447	11-Dodecenal	tr	0.00 ± 0.00	tr	0.00 ± 0.00		
		**Σ Aldehydes**	**140563.11 ± 13853.25**	**161396.39 ± 16528.83**	**178596.64 ± 13634.56**	**142758.91 ± 32936.18**		
**Alcohols**	1168	(2*E*)-2-Nonen-1-ol	tr	tr	tr	tr		
	1174	1-Nonanol	5796.65 ± 1808.49	5674.48 ± 1756.38	3554.22 ± 1689.16	2584.87 ± 698.17		
	1241	1-Decanol	tr	tr	tr	1925.27 ± 355.17		
	1278	(2*E*)-2-Decen-1-ol	4394.27 ± 735.42	5769.61 ± 603.15	5008.62 ± 304.86	4695.48 ± 1046.17		
	1476	1-Dodecanol	3650.76 ± 592.98	tr	938.50 ± 614.16	3081.67 ± 636.60		
	1691	1-Tetradecanol	870160.00 ± 154751.37	881215.51 ± 631419.54	434888.64 ± 125005.90	1012466.80 ± 696563.51		
		**Σ Alcohols**	**884449.51 ± 155908.33**	**894829.30 ± 633318.68**	**444808.79 ± 127744.82**	**1024961.32 ± 696486.15**		
**Ketones**	1118	2-Methyl-6-methylene-1,7-octadien-3-one	864.20 ± 735.22	6479.56 ± 983.50	6529.11 ± 670.54	9386.42 ± 1366.83		
		**Σ Ketones**	**864.20 ± 735.22**	**6479.56 ± 983.50**	**6529.11 ± 670.54**	**9386.42 ± 1366.83**		
**Esters**	1010	(4*E*)-4-Hexen-1-yl acetate	tr	0.00 ± 0.00	tr	0.00 ± 0.00		
	1015	Hexyl acetate	4317.00 ± 2173.94	0.00 ± 0.00	tr	0.00 ± 0.00		
		**Σ Esters**	**4320.31 ± 2175.94**	**0.00 ± 0.00**	**tr**	**0.00 ± 0.00**		
**Monoterpenes hydrocarbons**	992	*β*-myrcene	145269.05 ± 31647.94 a	16778.05 ± 4457.39 bc	19931.71 ± 1832.88 b	13945.09 ± 3717.64 c	23.74	<0.001
	1029	Limonene	84227.91 ± 13791.24	20623.79 ± 1686.47	31499.13 ± 1651.19	17478.59 ± 1066.74	52.34	<0.0001

Chemical classes	LRI	Compound	Peak Area (Mean ± SE)				$F_{3,8}$	*P* value
			HI-Black	HI-Green	LI-Black	LI-Green		
			a	bc	b	c		
	1048	(*E*)-*β*-ocimene	28556.75 ± 8243.12 a	15618.71 ± 3976.05 ab	13554.19 ± 2094.72 ab	9787.34 ± 1555.76 b	4.01	0.049
	1059	*γ*-terpinene	0.00 ± 0.00	0.00 ± 0.00	2227.57 ± 1196.77	tr		
		**Σ Monoterpenes hydrocarbons**	**258053.71 ± 49333.47 a**	**53020.55 ± 4447.76 bc**	**67212.60 ± 5800.25 ab**	**41214.36 ± 5220.71 c**	**40.20**	**<.0001**
**Monoterpenes oxigenated**	1192	*α*-terpineol	tr	7697.56 ± 4617.93	12341.51 ± 3627.22	5854.44 ± 2300.42		
	1371	Limonene diepoxide	tr b	4086.67 ± 1514.68 a	2841.46 ± 439.56 ab	3985.08 ± 761.55 a	12.21	0.002
		**Σ Monoterpenes oxigenated**	**tr**	**11784.23 ± 4872.34**	**15182.97 ± 3225.78**	**9839.52 ± 2809.47**		
**Sesquiterpenes hydrocarbons**	1365	Cyclosativene	5654.52 ± 932.89	tr	3871.45 ± 852.72	3018.55 ± 996.66		
	1368	Longicyclene	tr	tr	tr	tr		
	1376	*α*-copaene	17192.78 ± 2584.12	7191.30 ± 860.37	12343.52 ± 2018.33	11987.54 ± 2888.54		
	1420	*β*-caryophyllene	5465.63 ± 744.01	tr	2929.26 ± 939.46	tr		
	1429	*β*-copaene	1307.29 ± 679.67	984.51 ± 557.02	tr	tr		
	1458	(*E*)-*β*-farnesene	0.00 ± 0.00 c	0.00 ± 0.00 c	tr b	2646.53 ± 813.31 a	147.97	<.0001
	1486	*β*-selinene	1547,74 ± 782.10	2583.94 ± 1287.00	1920.11 ± 1201.54	1328.76 ± 344.22		
	1489	Valencene	10759.46 ± 4675.21	11057.14 ± 1454.44	11293.00 ± 2373.18	5348.90 ± 1133.42		
	1496	*α*-selinene	3170.44 ± 658.87	3894.70 ± 882.56	3893.33 ± 914.19	2002.83 ± 333.60		
	1502	*α*-muurulene	2399.47 ± 667.91	tr	2376.68 ± 155.96	2055.82 ± 287.59		

Chemical classes	LRI	Compound	Peak Area (Mean ± SE)				$F_{3,8}$	*P* value
			HI-Black	HI-Green	LI-Black	LI-Green		
	1510	(*E*,*E*)-*α*-farnesene	15678.45 ± 7845.87 ab	4237.22 ± 284.29 b	25332.58 ± 8383.25 a	4779.75 ± 498.85 b	6.54	0.015
		**Σ Sesquiterpenes hydrocarbons**	**63505.04 ± 11278.38 a**	**31239.70 ± 4999.03 b**	**64724.14 ± 12777.64 a**	**34976.61 ± 4158.86 b**	**5.38**	**0.025**
**Sesquiterpenes oxigenated**	1619	Epi-cedrol	4037.34 ± 1845.72	tr	tr	2035.13 ± 1164.65		
	1664	*α*-bisabolol	5135.23 ± 3053.60	3822.11 ± 459.57	6482.98 ± 499.04	4512.21 ± 1335.45		
	1842	Farnesol acetate	5747.11 ± 1955.90 b	6177.53 ± 1573.94 b	14396.48 ± 2574.07 a	19916.63 ± 9357.18 a	4.45	0.041
		**Σ Sesquiterpenes oxigenated**	**14923.01 ± 3877.17**	**10414.33 ± 1073.79**	**21535,58 ± 3473.19**	**26463.98 ± 8560.10**		
**Apocarotenoids**	1455	Geranylacetone	12399.69 ± 4419.98 b	41979.24 ± 17383.12 ab	76389.37 ± 26045.83 a	75846.31 ± 34372.79 a	4.83	0.033
		**Σ Apocarotenoids**	**12399.69 ± 4419.98 b**	**41979.24 ± 17383.12 ab**	**76389.37 ± 26045.83 a**	**75846.31 ± 34372.79 a**	**4.83**	**0.033**

Supplementary Table S5 Principal components identified after Principal Component Analysis (PCA) of volatile emissions from three olive cultivars. Bolded components were statistically analysed via General Linear Model to determine source of variation

**Number**	**Eigenvalue**	**Percentage**	**Cumulative Percentage**
**1**	**13,2820**	**22,512**	**22,512**
**2**	**7,5334**	**12,768**	**35,280**
**3**	**6,0074**	**10,182**	**45,462**
**4**	**5,2070**	**8,825**	**54,288**
**5**	**3,0944**	**5,245**	**59,533**
**6**	**2,5418**	**4,308**	**63,841**
**7**	**2,4357**	**4,128**	**67,969**
**8**	**2,2304**	**3,780**	**71,749**
9	1,7825	3,021	74,770
10	1,6981	2,878	77,649
11	1,5665	2,655	80,304
12	1,3734	2,328	82,631
13	1,2386	2,099	84,731
14	1,0884	1,845	86,576
15	1,0049	1,703	88,279
16	0,9628	1,632	89,911
17	0,8781	1,488	91,399
18	0,7782	1,319	92,718
19	0,5707	0,967	93,685
20	0,5476	0,928	94,613
21	0,4045	0,686	95,299
22	0,3922	0,665	95,964
23	0,3508	0,595	96,558
24	0,2967	0,503	97,061
25	0,2858	0,484	97,545
26	0,2802	0,475	98,020
27	0,2229	0,378	98,398
28	0,2035	0,345	98,743
29	0,1684	0,285	99,029
30	0,1521	0,258	99,286
31	0,1317	0,223	99,510
32	0,0922	0,156	99,666
33	0,0768	0,130	99,796
34	0,0649	0,110	99,906
35	0,0554	0,094	100,000

Supplementary Table S6. Eigenvectors of every volatile compound emitted by olive fruits for 8 selected Principal Components

Compound	PC1	PC2	PC3	PC4	PC5	PC6	PC7	PC8
β-myrcene	-0.01272	0.02405	-0.05211	0.34525	-0.08204	-0.08621	-0.04414	-0.0618
(4E)-4-Hexen-1-yl acetate	-0.0451	0.01844	-0.23911	-0.01995	0.28715	0.14919	0.03729	-0.09251
Hexyl acetate	-0.09941	-0.04145	-0.09078	-0.017	0.17193	0.35663	-0.19529	-0.18702
α-terpinene	0.2047	-0.20063	0.03776	0.00669	-0.11766	-0.06985	-0.05717	-0.03143
o-cymene	0.21002	-0.1915	0.03141	0.00188	-0.10673	-0.07419	-0.05969	-0.04894
Limonene	0.16609	-0.0422	-0.02404	0.25759	-0.08373	0.1765	-0.07687	-0.08441
(*E*)-*β*-ocimene	0.14307	0.02115	-0.08673	0.25322	-0.09795	-0.03483	-0.0486	-0.19296
γ-terpinene	0.2169	-0.11328	0.01811	0.0145	-0.06455	0.02389	-0.09433	-0.02085
α-terpinolene	0.18771	-0.21116	0.00418	0.02493	-0.09375	-0.14143	0.00191	0.01985
n-undecane	0.01672	-0.15433	-0.08808	0.18605	0.08774	-0.16316	-0.13906	0.12483
Nonanal	0.21288	-0.14783	-0.12755	0.04653	0.02222	0.05957	-0.10323	0.01172
2-Methyl-6-methylene-1,7-octadien-3-one	0.02692	0.15912	0.09789	0.01284	0.30161	-0.15356	-0.16177	-0.24081
trans-alloocimene	0.17926	-0.12707	0.05318	-0.0882	0.02007	-0.23216	-0.02026	-0.04187
(2*E*)-2-Nonen-1-ol	-0.02267	0.057	-0.27481	0.12628	-0.05311	-0.08014	0.06625	-0.10679
1-Nonanol	-0.10426	-0.1087	0.02926	0.09415	-0.10221	0.05448	0.01201	0.1498
4-terpineol	0.19206	-0.0738	0.11598	0.04636	0.17575	-0.15847	0.0206	-0.14801
α-terpineol	0.06258	-0.08591	-0.01814	0.1451	0.00181	0.23086	0.18746	0.02476
n-dodecane	0.13934	-0.22811	-0.02845	0.16504	0.1915	-0.07048	-0.0278	0.06256
Decanal	0.16665	0.18289	0.08356	0.05031	-0.11988	0.17165	-0.03225	-0.07727
1-Decanol	-0.02938	0.00615	0.22121	0.0884	0.03099	-0.27791	0.15214	-0.054
(2*E*)-2-Decen-1-ol	0.02191	0.09917	0.03962	-0.01094	0.19497	0.14111	0.35777	0.00186
10-Undecenal	0.2103	0.04759	-0.20418	-0.0886	-0.07127	0.0497	0.03762	0.00893
n-tridecane	0.06244	-0.1886	0.10457	0.2574	0.03541	0.01493	0.21633	-0.01882
Undecanal	0.17201	0.07975	0.02762	0.04072	0.02005	0.12754	0.20129	0.03015
(6*Z*)-6-Tridecene	-0.01148	-0.10329	-0.18331	0.05369	-0.09035	-0.11484	0.28161	0.29971
α-longipinene	0.12568	-0.03089	-0.22233	-0.02495	-0.18997	0.04512	-0.1177	0.01105
Cyclosativene	-0.00328	0.18571	-0.23009	0.18767	-0.03534	-0.09655	-0.00424	0.06203
Longicyclene	-0.01256	0.18711	-0.13354	0.11932	0.14728	-0.00839	-0.04003	-0.0267
Limonene diepoxide	0.09515	0.05739	0.12464	-0.09718	-0.04264	0.17191	0.30798	-0.03206
α-copaene	0.01212	0.2039	-0.19317	0.18591	-0.03139	0.15802	-0.04567	0.12962
Isolongifolene	0.20214	-0.14374	0.10622	-0.02608	0.08827	0.01843	0.07327	-0.01328
(4*E*)-4-Tetradecene	-0.0841	-0.14912	-0.08876	0.17033	0.12636	0.18413	-0.09819	0.26153
n-tetradecane	-0.02462	-0.1332	-0.13909	0.04062	0.35079	-0.08981	0.02665	0.14949
Dodecanal	0.19325	0.13835	-0.00081	-0.03282	0.02509	0.06648	0.14844	0.04275
(*E*)-β-caryophyllene	0.12193	-0.05719	0.01631	-0.03603	0.06048	0.01936	-0.18249	0.15944
β-copaene	0.04719	0.09411	0.04168	-0.02958	0.26766	-0.10825	-0.00883	0.36911
(*Z*)-*α*-bergamotene	0.184	-0.15034	0.07129	-0.10866	0.0806	0.17415	-0.05852	0.07149

**Compound**	**PC1**	**PC2**	**PC3**	**PC4**	**PC5**	**PC6**	**PC7**	**PC8**
11-Dodecenal	-0.03645	-0.18743	-0.02688	0.06748	0.17671	0.29275	0.06978	-0.10191
Geranylacetone	0.14499	0.19333	0.08675	0.05469	-0.03233	0.05021	0.19833	-0.03037
(*E*)-*β*-farnesene	0.02929	0.08312	0.24503	0.06812	0.02849	0.1741	0.17185	0.03026
1-Dodecanol	-0.00292	0.02708	0.21929	0.16048	-0.1649	0.10059	-0.13234	0.30569
β-selinene	0.01323	0.16455	0.11082	0.06172	0.0809	-0.1275	-0.0692	0.18814
Valencene	0.19498	0.10041	0.06993	0.13924	-0.03252	-0.02516	-0.10321	-0.0197
α-selinene	0.04456	0.21551	0.09642	0.16072	-0.02028	0.02548	-0.11457	-0.00762
n-pentadecane	-0.04468	0.00562	-0.04287	0.32164	-0.04047	0.0521	0.02935	-0.09492
α-muurulene	0.06277	0.23838	0.04434	0.19144	0.10664	-0.20838	0.04191	0.12381
(*E,E*)-*α*-farnesene	0.22298	0.01748	0.0391	-0.00343	0.05719	0.05829	-0.09733	-0.04267
Dihydro-*β*-agarofuran	0.20024	0.15076	-0.14216	-0.11052	0.11549	-0.05787	-0.00715	-0.06077
Longipinanol	0.2002	0.151	-0.14227	-0.11013	0.11497	-0.05674	-0.00637	-0.05671
n-hexadecane	-0.16066	-0.0272	0.16861	0.08245	0.12529	-0.03705	-0.10466	-0.18565
Tetradecanal	0.22038	0.00592	-0.12326	-0.04236	-0.05574	0.02809	0.09627	0.06649
Epi-cedrol	0.04876	0.07948	0.09736	0.09274	0.22717	0.03217	-0.07317	0.16938
α-bisabolol	-0.07993	0.00245	0.06164	0.04379	-0.1594	-0.1253	0.16187	-0.01888
1-Tetradecanol	0.0774	0.11472	0.1443	-0.03766	-0.01559	0.18733	-0.27277	0.27711
n-heptadecane	-0.03491	-0.01526	0.00627	0.33447	0.06795	-0.01266	0.06396	-0.1715
n-octadecane	0.01743	0.09372	0.2671	0.01101	-0.04489	0.06836	-0.23996	-0.15869
Farnesol acetate	0.13364	0.16413	0.11697	0.0193	-0.11208	0.00802	0.03461	0.09301
n-nonadecane	0.06867	-0.14259	0.24264	0.0521	0.21724	0.03105	0.09369	-0.01195

Supplementary Table S7. Correlations between volatiles from olive fruits and factors identified after Multi-Factorial Analysis (MFA). Bolded values represent highly correlation between the chemical and the factor

**Compound**	**Factor 1**	**Factor 2**	**Factor 3**	**Factor 4**	**Factor 5**	**Factor 6**	**Factor 7**	**Factor 8**
β-myrcene	-0,009783	-0,129079	**0,803190**	0,060148	-0,132465	-0,034401	0,027244	-0,107219
(4*E*)-4-Hexen-1-yl acetate	-0,280132	0,251719	0,089807	**-0,523067**	0,015067	0,026791	0,067807	**0,529413**
Hexyl acetate	-0,243714	-0,149093	0,002083	-0,108836	-0,081382	0,212764	-0,160678	**0,780820**
α-terpinene	**0,921179**	0,145701	-0,011852	0,094302	-0,062902	-0,103197	-0,180211	-0,089663
o-cymene	**0,916706**	0,185094	-0,009380	0,083598	-0,062217	-0,075201	-0,180393	-0,087010
Limonene	**0,529104**	0,200388	**0,607035**	0,312465	0,108072	-0,073634	-0,083015	0,222982
(*E*)-*β*-ocimene	0,355506	0,344252	**0,699994**	0,127509	-0,036653	0,043882	-0,139395	-0,044845
γ-terpinene	**0,783227**	0,310399	0,034659	0,202357	-0,003031	-0,047658	-0,072327	0,046235
α-terpinolene	**0,886473**	0,120010	0,025830	-0,050294	-0,079895	-0,189931	-0,112937	-0,165823
n-undecane	0,368793	-0,190731	0,352385	-0,258355	-0,364298	-0,154441	0,245027	0,058543
Nonanal	**0,771031**	0,410663	0,154316	-0,043837	-0,106497	-0,192170	-0,020272	0,273530
2-Methyl-6-methylene-1,7-octadien-3-one	-0,087071	0,213736	0,080772	-0,022607	0,005693	**0,787767**	0,329732	-0,060494
trans-alloocimene	**0,741353**	0,244684	-0,204197	-0,094693	-0,063135	0,108956	0,005252	-0,270312
(2*E*)-2-Nonen-1-ol	-0,267271	0,311953	0,492001	-0,328096	-0,201417	-0,201715	-0,171434	0,008414
1-Nonanol	-0,061914	**-0,518056**	0,083581	0,020592	-0,074465	-0,294082	-0,036678	0,044144
4-terpineol	**0,733874**	0,212775	0,082615	-0,079226	0,203851	0,373632	0,160860	-0,168260
α-terpineol	0,270248	-0,050192	0,288415	-0,007822	0,385289	-0,269617	-0,065743	0,239579
n-dodecane	**0,818358**	-0,056900	0,280185	-0,297166	-0,024429	-0,079888	0,246129	0,161217
Decanal	0,127663	**0,504896**	0,183620	**0,626687**	0,303320	0,142487	-0,019766	-0,028626
1-Decanol	0,064947	-0,357625	0,086714	-0,031622	0,209108	0,292759	0,144945	**-0,563462**
(2*E*)-2-Decen-1-ol	-0,169559	0,157708	-0,024724	-0,153295	**0,677972**	0,030165	0,177132	0,029360
10-Undecenal	0,321255	**0,852715**	-0,005747	0,020757	0,013122	-0,269532	-0,108227	0,078004
n-tridecane	**0,571528**	-0,392081	0,441818	-0,125481	0,378051	-0,110102	0,009895	-0,056352
Undecanal	0,273891	0,445724	0,121186	0,193083	**0,502520**	-0,084877	0,105778	-0,029219
(6*Z*)-6-Tridecene	0,038512	-0,017311	0,109955	-0,396687	-0,009271	**-0,738228**	0,049530	-0,180373
α-longipinene	0,264095	**0,521694**	0,102981	0,068548	-0,311673	-0,353245	-0,258459	0,148609
Cyclosativene	-0,398964	0,415815	**0,587366**	-0,052665	-0,200263	-0,204215	0,171889	-0,079927
Longicyclene	-0,387352	0,336153	0,388193	-0,072894	-0,039007	0,125995	0,269328	0,104524
Limonene diepoxide	0,104556	0,207017	-0,225665	0,194838	**0,656625**	-0,030096	-0,122891	-0,108587
α-copaene	-0,409537	0,407189	**0,535164**	0,207482	-0,038404	-0,267889	0,207497	0,221545
Isolongifolene	**0,818445**	0,159894	-0,130210	0,014746	0,299166	0,053651	0,055784	0,004886
(4*E*)-4-Tetradecene	0,031655	-0,444239	0,236069	-0,158650	-0,154211	-0,367494	0,275348	**0,507522**

**Compound**	**Factor 1**	**Factor 2**	**Factor 3**	**Factor 4**	**Factor 5**	**Factor 6**	**Factor 7**	**Factor 8**
n-tetradecane	0,146827	-0,097487	0,037849	**-0,649031**	-0,111278	-0,102136	0,415747	0,264509
Dodecanal	0,212935	**0,655700**	0,002963	0,215997	0,400546	-0,035984	0,157301	-0,091124
(*E*)-*β*-caryophyllene	0,445684	0,175014	-0,145472	0,162671	-0,138082	-0,048235	0,247838	0,144401
β-copaene	0,000022	0,163403	-0,187118	-0,019508	0,067260	-0,037556	**0,770318**	-0,073563
(*Z*)-*α*-bergamotene	**0,741721**	0,175648	-0,329855	0,136250	0,174708	-0,041524	0,055399	0,291910
11-Dodecenal	0,196625	-0,340190	0,089286	-0,295545	0,238590	-0,023580	-0,130106	**0,579424**
Geranylacetone	0,043778	0,471201	0,178888	0,347219	**0,518487**	0,087276	0,109585	-0,253255
(*E*)-*β*-farnesene	0,008440	-0,168324	0,019351	0,387101	**0,607854**	0,147968	0,158971	-0,080790
1-Dodecanol	0,074750	-0,404003	0,130295	**0,689662**	0,039630	-0,176194	0,292000	-0,101309
β-selinene	-0,157042	0,063039	0,061181	0,269796	0,017997	0,143204	**0,510760**	-0,255401
Valencene	0,409528	0,421966	0,338237	0,437592	0,096420	0,161362	0,191759	-0,138416
α-selinene	-0,173733	0,183825	0,369609	**0,501538**	0,092955	0,251018	0,254668	-0,113967
n-pentadecane	-0,091149	-0,223632	**0,736262**	0,010219	0,043100	-0,040417	-0,046632	0,066734
α-muurulene	-0,166361	0,304024	0,437972	0,175236	0,132715	0,153546	**0,571239**	-0,396154
(*E*,*E*)-*α*-farnesene	**0,575894**	0,499176	0,023706	0,266807	0,132324	0,162177	0,119707	0,084204
Dihydro-*β*-agarofuran	0,175805	**0,934600**	-0,047547	-0,033699	0,047493	0,122126	0,148074	-0,007842
Longipinanol	0,174889	**0,934013**	-0,047880	-0,031809	0,048526	0,116918	0,151282	-0,007474
n-hexadecane	-0,232361	**-0,631890**	0,080082	-0,053051	-0,038183	0,495358	0,016989	0,024488
Tetradecanal	0,465795	**0,683974**	0,015171	0,045482	0,136566	-0,289624	-0,015816	-0,011471
Epi-cedrol	0,072111	0,026999	0,095887	0,160649	0,153396	0,162107	**0,566657**	0,091691
α-bisabolol	-0,187965	-0,278423	0,074838	-0,012366	0,068908	-0,088193	-0,171959	-0,374366
1-Tetradecanol	0,065775	0,119836	-0,214995	**0,704988**	-0,023120	0,012387	0,402248	0,170947
n-heptadecane	0,012292	-0,276343	**0,747367**	-0,118493	0,132326	0,153963	0,019825	0,015585
n-octadecane	0,038267	-0,151358	-0,052009	**0,611446**	0,048542	**0,554871**	0,002256	-0,052272
Farnesol acetate	0,098907	0,361543	0,038669	**0,519818**	0,255129	0,012501	0,166121	-0,283702
n-nonadecane	**0,558258**	-0,357404	-0,084688	-0,029051	0,422642	0,270522	0,232826	0,019340

Supplementary Table S8 Effect tests of General Linear Models on Factors obtained after Multi-Factorial analysis (MFA) respect to the main variable (Cultivar, Infestation Status and Maturation) and their interaction

**Response Factor1 "cv. Ottobratica & cv. Sinopolese" Effect Tests**					
**Source**	**Nparm**	**DF**	**Sum of Squares**	**F Ratio**	**Prob > F**
Cultivar	2	2	10.736	70.89	<.0001*
Infestation Status	1	1	13.408	177.05	<.0001*
Cultivar*Infestation Status	2	2	4.298	28.38	<.0001*
Maturation	1	1	0.013	0.18	0.6780
Cultivar*Maturation	2	2	2.983	19.69	<.0001*
Infestation Status*Maturation	1	1	0.086	1.14	0.2969
Cultivar*Infestation Status*Maturation	2	2	1.657	10.94	0.0004*
**Response Factor2 "cv. Ottobratica" Effect Tests**					
**Source**	**Nparm**	**DF**	**Sum of Squares**	**F Ratio**	**Prob > F**
Cultivar	2	2	10.057	72.18	<.0001*
Infestation Status	1	1	2.301	33.04	<.0001*
Cultivar*Infestation Status	2	2	8.187	58.77	<.0001*
Maturation	1	1	2.005	28.78	<.0001*
Cultivar*Maturation	2	2	4.874	34.98	<.0001*
Infestation Status*Maturation	1	1	1.978	28.40	<.0001*
Cultivar*Infestation Status*Maturation	2	2	3.926	28.18	<.0001*
**Response Factor3 "Infestation status" Effect Tests**					
**Source**	**Nparm**	**DF**	**Sum of Squares**	**F Ratio**	**Prob > F**
Cultivar	2	2	0.154	0.30	0.5881
Infestation Status	1	1	13.445	13.14	0.0001*
Cultivar*Infestation Status	2	2	5.542	5.41	0.0115*
Maturation	1	1	0.007	0.01	0.9079
Cultivar*Maturation	2	2	2.027	1.98	0.1598

Infestation Status*Maturation	1	1	0.139	0.27	0.6068
Cultivar*Infestation Status*Maturation	2	2	1.404	1.37	0.2727
**Response Factor4 "cv. Roggianella" Effect Tests**					
**Source**	**Nparm**	**DF**	**Sum of Squares**	**F Ratio**	**Prob > F**
Cultivar	2	2	13.339	24.88	<.0001*
Infestation Status	1	1	3.191	11.91	0.0021*
Cultivar*Infestation Status	2	2	9.336	17.42	<.0001*
Maturation	1	1	1.185	4.42	0.0462*
Cultivar*Maturation	2	2	1.254	2.34	0.1180
Infestation Status*Maturation	1	1	0.180	0.67	0.4208
Cultivar*Infestation Status*Maturation	2	2	0.084	0.16	0.8561
**Response Factor5 "Infestation status cv. Roggianella" Effect Tests**					
**Source**	**Nparm**	**DF**	**Sum of Squares**	**F Ratio**	**Prob > F**
Cultivar	2	2	4.216	3.54	0.0450*
Infestation Status	1	1	2.881	4.84	0.0377*
Cultivar*Infestation Status	2	2	3.087	2.59	0.0958
Maturation	1	1	2.512	4.22	0.0511
Cultivar*Maturation	2	2	1.827	1.53	0.2363
Infestation Status*Maturation	1	1	5.652	9.49	0.0051*
Cultivar*Infestation Status*Maturation	2	2	0.528	0.44	0.6470
**Response Factor6 "Infestation status*Maturation" Effect Tests**					
**Source**	**Nparm**	**DF**	**Sum of Squares**	**F Ratio**	**Prob > F**
Cultivar	2	2	3.565	2.18	0.1350
Infestation Status	1	1	0.032	0.04	0.8443
Cultivar*Infestation Status	2	2	0.537	0.33	0.7236
Maturation	1	1	0.789	0.96	0.3360

Cultivar*Maturation	2	2	0.741	0.45	0.6409
Infestation Status*Maturation	1	1	5.389	6.59	0.0169*
Cultivar*Infestation Status*Maturation	2	2	4.314	2.64	0.0922
**Response Factor7 "Green" Effect Tests**					
**Source**	**Nparm**	**DF**	**Sum of Squares**	**F Ratio**	**Prob > F**
Cultivar	2	2	1.563	1.44	0.2575
Infestation Status	1	1	0.002	0.003	0.9529
Cultivar*Infestation Status	2	2	0.924	0.85	0.4403
Maturation	1	1	2.938	5.40	0.0289*
Cultivar*Maturation	2	2	13.263	12.19	0.0002*
Infestation Status*Maturation	1	1	1.901	3.49	0.0739
Cultivar*Infestation Status*Maturation	2	2	1.352	1.24	0.3066
**Response Factor8 "Black" Effect Tests**					
**Source**	**Nparm**	**DF**	**Sum of Squares**	**F Ratio**	**Prob > F**
Cultivar	2	2	3.327	3.04	0.0665
Infestation Status	1	1	0.024	0.04	0.8343
Cultivar*Infestation Status	2	2	1.004	0.92	0.4131
Maturation	1	1	15.185	27.76	<.0001*
Cultivar*Maturation	2	2	0.588	0.54	0.5914
Infestation Status*Maturation	1	1	0.832	1.52	0.2294
Cultivar*Infestation Status*Maturation	2	2	0.909	0.83	0.4478

Supplementary Figure S1. Principal Component Analysis (PCA) score plots showing clusters of volatiles related to infestation status within each cultivar: a) cv. Ottobratica, b) cv. Roggianella, c) cv. Sinopolese.

● High-infestation (HI) status; ○ Low-infestation (LI) status.

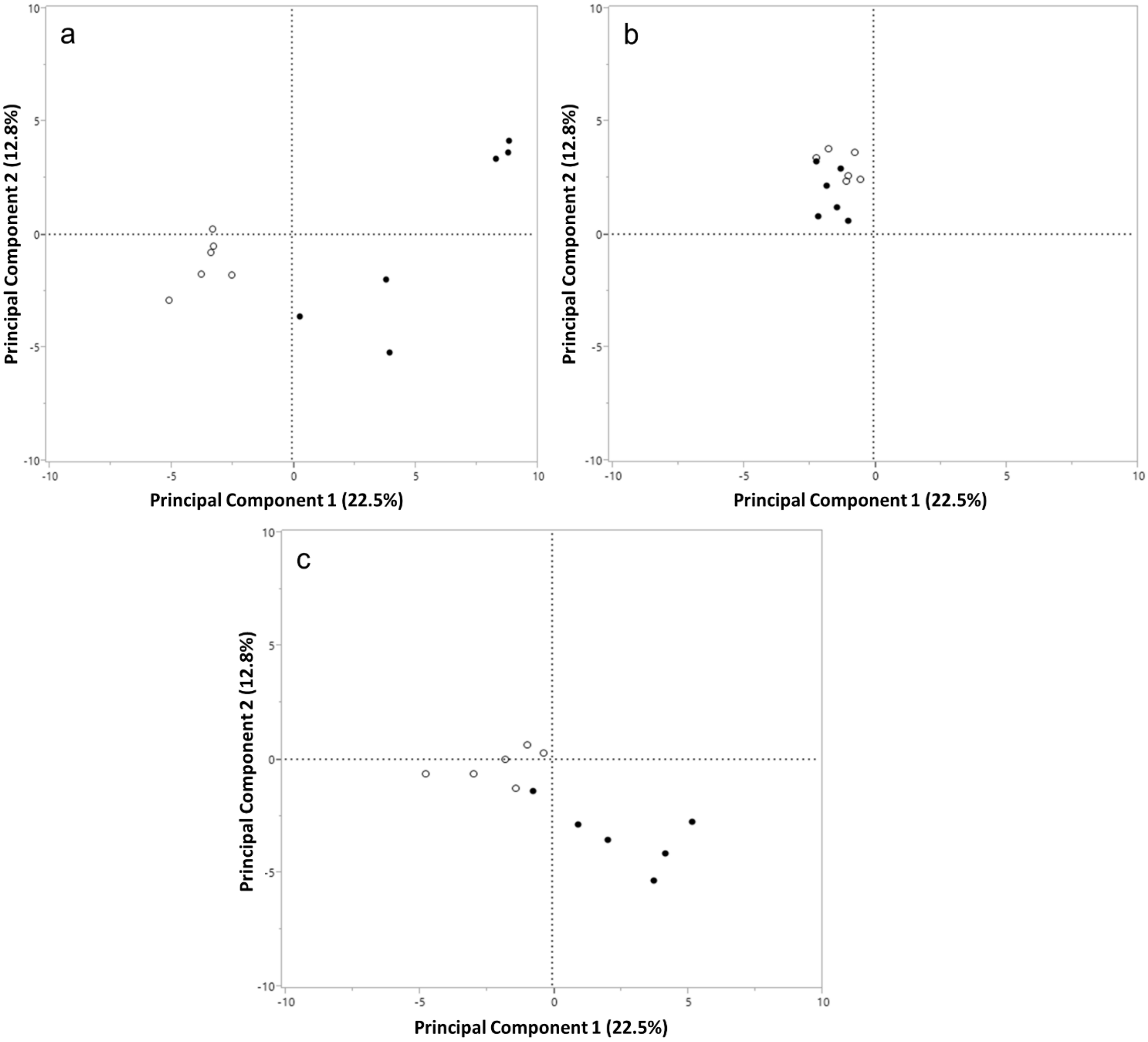

Supplement: Supplementary file 1 — Supplementary Information. [file 41598_2020_58379_MOESM1_ESM.pdf]
